# Low-impedance tissue-device interface using homogeneously conductive hydrogels chemically bonded to stretchable bioelectronics

**DOI:** 10.1126/sciadv.adi7724

**Published:** 2024-03-20

**Authors:** Yoonsoo Shin, Hyun Su Lee, Yongseok Joseph Hong, Sung-Hyuk Sunwoo, Ok Kyu Park, Sueng Hong Choi, Dae-Hyeong Kim, Sangkyu Lee

**Affiliations:** ^1^ Center for Nanoparticle Research, Institute for Basic Science (IBS), Seoul 08826, Republic of Korea.; ^2^ School of Chemical and Biological Engineering, Institute of Chemical Processes, Seoul National University, Seoul 08826, Republic of Korea.; ^3^ Department of Radiology, Seoul National University College of Medicine, Seoul 03080, Republic of Korea.; ^4^ Department of Materials Science and Engineering, Seoul National University, Seoul 08826, Republic of Korea.

## Abstract

Stretchable bioelectronics has notably contributed to the advancement of continuous health monitoring and point-of-care type health care. However, microscale nonconformal contact and locally dehydrated interface limit performance, especially in dynamic environments. Therefore, hydrogels can be a promising interfacial material for the stretchable bioelectronics due to their unique advantages including tissue-like softness, water-rich property, and biocompatibility. However, there are still practical challenges in terms of their electrical performance, material homogeneity, and monolithic integration with stretchable devices. Here, we report the synthesis of a homogeneously conductive polyacrylamide hydrogel with an exceptionally low impedance (~21 ohms) and a reasonably high conductivity (~24 S/cm) by incorporating polyaniline-decorated poly(3,4-ethylenedioxythiophene:polystyrene). We also establish robust adhesion (interfacial toughness: ~296.7 J/m^2^) and reliable integration between the conductive hydrogel and the stretchable device through on-device polymerization as well as covalent and hydrogen bonding. These strategies enable the fabrication of a stretchable multichannel sensor array for the high-quality on-skin impedance and pH measurements under in vitro and in vivo circumstances.

## Teaser

A high-performance stretchable sensor array is implemented by the monolithic integration of a homogeneously conductive hydrogel.

## INTRODUCTION

Flexible and stretchable bioelectronics, with distinct advantages in terms of biointegration, has brought many opportunities to emerging biorelated fields such as wearable bioelectronics (*1*–*3*), bioinspired robotics (*4*, *5*), and medical implants (*6*, *7*). In particular, stretchable bioelectronics has proved its potential for the next-generation point-of-care medical diagnostics and health care (*8*–*10*). Despite substantial progresses in the stretchable bioelectronics, including multifunctional sensor developments (*11*, *12*), stretchable device designs (*13*, *14*), and improved long-term user comfort (*15*–*17*), however, practical challenges still remain. One critical challenge is the microscale nonconformal contact between the device and the target tissue (*18*). Most of the stretchable devices have been fabricated with materials with high modulus, such as polymer films [polyimide: 2.5 GPa and parylene C: 3.2 GPa (*19*)], metals [gold: 70 GPa and copper: 119 GPa (*20*)], and oxide films [indium tin oxide: 89 GPa (*21*) and silicon dioxide: 65 GPa (*22*)]. Locally, these high-modulus materials exhibit nonconformal contacts on the rugged organ surface (*23*). The noncontacted areas often show a high contact impedance, which can be even more accelerated under dynamically mobile environments. Thereby, these locally nonconformal contacts and high-impedance interface substantially limit the performance of the stretchable bioelectronics (*24*–*26*).

Hydrogels feature tissue-like softness [elastic modulus: <100 kPa (*27*)] and water-rich property (*28*–*30*) and can also become electrically conductive by incorporating ions or conductive fillers (*31*, *32*). Thus, they can serve as an attractive interfacing medium between the stretchable bioelectronics and the human skin to overcome the microscale nonconformal contact, dry interface, and high contact impedance issues (*28*, *33*–*35*). However, there are critical difficulties in using conductive hydrogels as an interfacing medium with regard to their electrical performance, material homogeneity, and monolithic integration with the stretchable device.

Typically, hydrogels contain ions, exhibit poor conductivity, and consequently hamper efficient electrical signal transport. To prepare a conductive hydrogel enabling better ionic and electrical signal transport, a considerable amount of conducting materials such as metal-based materials [liquid metal (*32*), silver-based nanomaterials (*29*), or their mixture (*36*)] or conducting polymers [poly(3,4-ethylenedioxythiophene):poly(styrene sulfonate) (PEDOT:PSS) (*37*)] should be incorporated into the hydrogel matrix. Among them, metal-based hydrogels generally exhibit outstanding conductivity and stretchability, making them suitable for use as conductors in bioelectronics. Meanwhile, conducting polymer-based hydrogels can serve as an interfacing medium between the device and skin due to their higher softness and better homogeneity than metal-based ones. However, achieving material softness and homogeneity while incorporating high-concentration conducting fillers for high conductivity and low impedance is challenging (*38*, *39*). Further, water inside the hydrogel film creates a slippery interface (*40*) and restricts reliable integration of the hydrogel onto the surface of the stretchable bioelectronics (*41*). This poor integration can lead to unreliable device performance and difficulty in scaling up (*42*, *43*). Although methods for the use of tough hydrogels and the implementation of interfacial bonding have been reported (*44*, *45*), most studies have focused on the adhesion between hydrogels and tissues (*32*, *46*), and the adhesion between devices and conductive hydrogels has not been explored much.

## RESULTS

### Soft low-impedance tissue-device interface using conductive hydrogels chemically bonded to stretchable bioelectronics

**Fig. 1.**
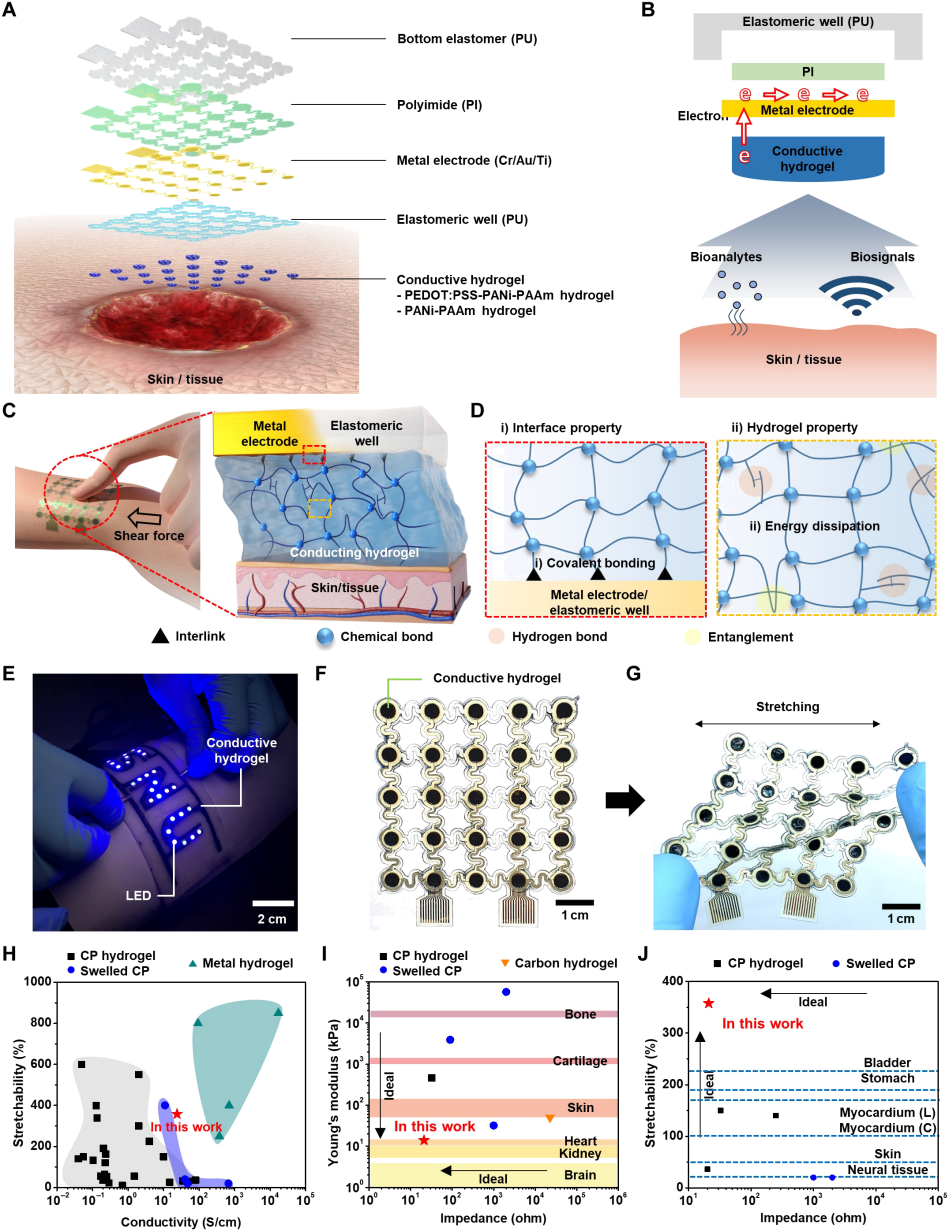
Seamless low-impedance tissue-device interface using homogeneously conductive hydrogels integrated with stretchable bioelectronics. (**A**) Exploded view of the stretchable bioelectronics integrated with the conductive hydrogels for the seamless and low-impedance tissue-device interface. (**B**) Cross-sectional view showing the role of the conductive hydrogel as an interfacing material. It facilitates the efficient transport of bioanalytes and biosignals between the tissue and the bioelectronics. (**C**) Reliable and monolithic adhesion between the conductive hydrogel and the stretchable device. (**D**) Strong bonding through surface covalent bonding and bulk hydrogen bonding. (**E**) Conductive hydrogel with sufficient conductivity to turn on a wearable light-emitting diode array. (**F**) Stretchable sensor array integrated with patterned conductive hydrogels. (**G**) Stretching deformation of the sensor array. (**H**) Conductivity-stretchability of conducting polymer–based hydrogel (CP hydrogel) (*37*, *38*, *64*–*70*), conducting polymer thin-film swelled by water (swelled CP) (*28*, *36*, *47*, *71*, *72*), and metal filler–incorporated hydrogel (metal hydrogel) (*29*, *73*, *74*). (**I** and **J**) Impedance–Young’s modulus (*28*, *42*, *47*, *66*, *75*) (I) and impedance-stretchability (*28*, *38*, *47*, *66*) (J) of CP hydrogel, swelled CP, and carbon material–based hydrogel (carbon hydrogel). Young’s moduli (*58**,* *76*) and stretchability (*72**,* *77*–*79*) corresponding to various organs (shown on the right side). “(L)” and “(C)” indicate the longitudinal and circumferential stretchabilities of myocardium, respectively.

To address the aforementioned challenges, we herein propose two strategies. First, we synthesize a homogeneously conductive, highly soft, and stretchable hydrogel with an exceptionally low impedance (21.2 to 22.9 ohms over the entire frequency range) and a reasonably high conductivity (~24 S/cm) by incorporating low-concentration PEDOT:PSS in the polyacrylamide (PAAm) hydrogel and subsequently decorating it with polyaniline (PANi). Figure 1A shows an exploded schematic illustration of the stretchable bioelectronics integrated with the conductive hydrogel that enables the seamless tissue-device interface. The ionically and electrically conductive hydrogel makes seamless, moisturized, and low-impedance contact to the target tissue including human skin. The conductive hydrogel is formed inside an elastomeric well made of polyurethane (PU) and electrically connected to the serpentine-shape stretchable metal electrode (Cr/Au/Ti) (Fig. 1B). The conductive hydrogel serves as an interfacing material that enables the facile transport of bioanalytes as well as electrical biosignals from the skin or the target tissue to the electrode.

Second, we establish reliable and monolithic adhesion between the conductive hydrogel and the stretchable device (Fig. 1C). To achieve this goal, we synthesize the conductive hydrogels inside the elastomeric PU wells on the stretchable multichannel electrode array in situ. In addition, the device surfaces (electrode surface and PU well surface) are modified with chemical anchoring groups, which form strong covalent bonds between the conductive hydrogel and the device surfaces (Fig. 1D, i). Furthermore, the conductive hydrogel on the device is treated in a bath containing a hydrogen-bonding agent to impart energy dissipative characteristics to the conductive hydrogel (Fig. 1D, ii), which enables reliable bonding of the hydrogel to the bioelectronics even under dynamic mechanical deformations through release of the induced stress.

The functionalized hydrogel shows reasonably high conductivity. For example, it is conductive enough to serve as a conductive interconnection for the light-emitting diode (LED) array (Fig. 1E). The materials and fabrication processes are also compatible with diverse system designs including the stretchable array design (Fig. 1F). Moreover, the soft and stretchable nature of the conductive hydrogel with strong adhesion and monolithic integration to the electrode allows the robust and reliable system construction even under dynamic mechanical deformations (Fig. 1G). To evaluate our hydrogel for use in stretchable bioelectronics, we compared its mechanical and electrical properties with those of previous studies (Fig. 1, H to J). While our hydrogel showed lower conductivity and stretchability compared to metal filler–incorporated hydrogels, it exhibited notable conductivity and stretchability among conducting polymer–based hydrogels (Fig. 1H). However, for a conductive hydrogel to serve as an interfacing material for transporting bioanalytes or electrical biosignals from the skin/tissue to the electrode, low impedance is crucial. Our developed hydrogel falls within the lowest range in terms of impedance and Young’s modulus (Fig. 1I). Moreover, its high stretchability makes it suitable for integration with stretchable bioelectronics (Fig. 1J). The resulting stretchable multichannel sensor array integrated with the conductive hydrogel, therefore, enables to measure impedance and pH reliably under both in vitro and in vivo circumstances (detailed results are shown in the later sections).

### Strategies for improving electrical properties of the conductive hydrogel

**Fig. 2.**
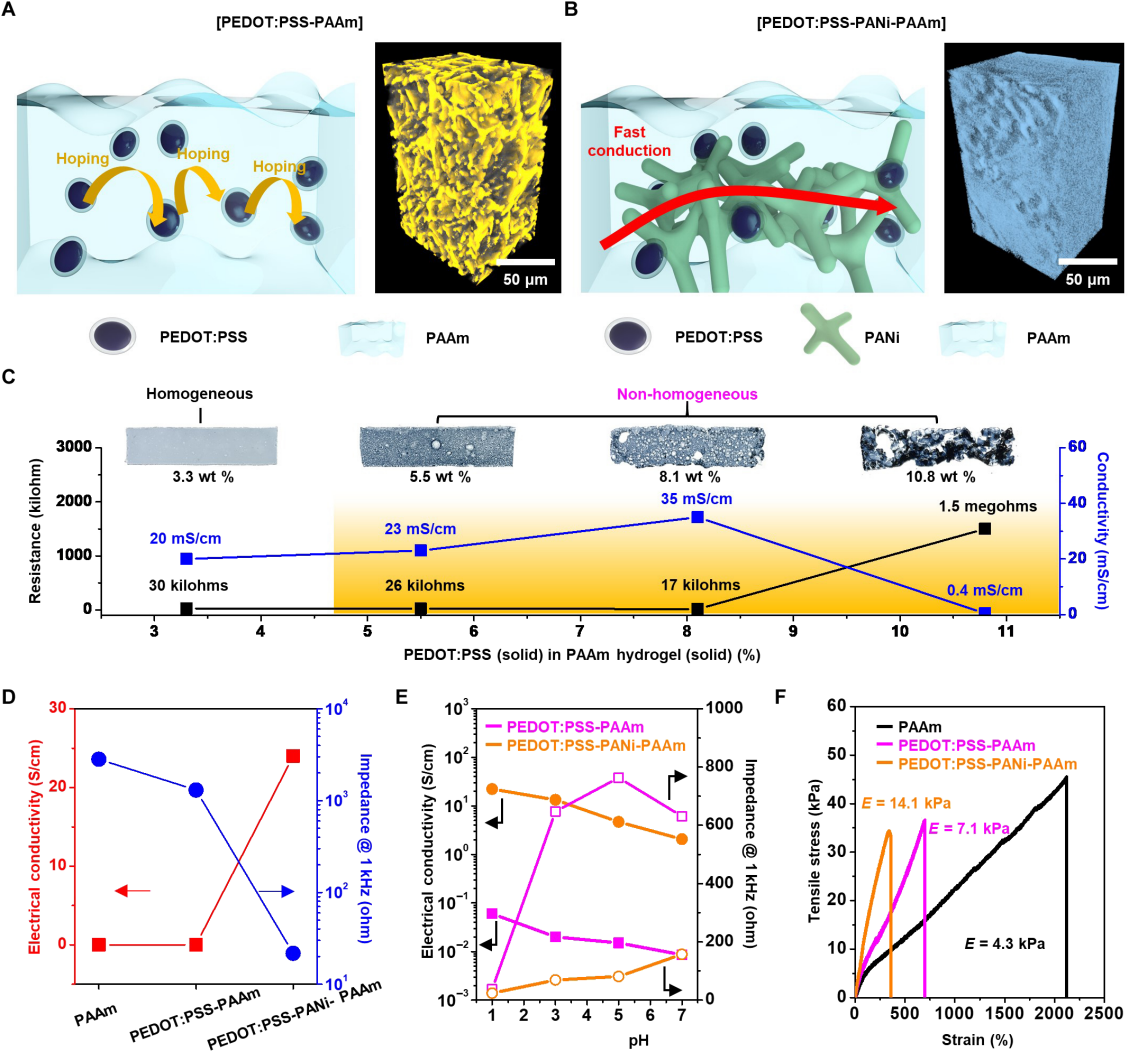
Homogeneously conductive hydrogel with tissue-like softness, low impedance, and reasonably high conductivity. (**A** and **B**) Schematic illustration of the conduction mechanism in PEDOT:PSS-PAAm hydrogel with a relatively low fraction of PEDOT:PSS (A) and PEDOT:PSS-PANi-PAAm hydrogel after treatment of the PEDOT:PSS-PAAm hydrogel with PANi (B). The inset images show the 3D x-ray tomograms of the freeze-dried hydrogels. (**C**) Images and electrical properties of PEDOT:PSS-PAAm hydrogels prepared with various concentrations of PEDOT:PSS. (**D**) Electrical conductivity and impedance for PAAm, PEDOT:PSS-PAAm, and PEDOT:PSS-PANi-PAAm hydrogels. (**E**) pH-dependent conductivity and impedance changes for PEDOT:PSS-PAAm and PEDOT:PSS-PANi-PAAm hydrogels. (**F**) Stress-strain curves of PAAm, PEDOT:PSS-PAAm, and PEDOT:PSS-PANi-PAAm hydrogels. *E* stands for the calculated modulus from the curve.

PAAm is a highly soft and stretchable hydrogel. However, its usage in electronic applications has been limited because of its low electrical conductivity. One method to improve the conductivity is to mix conducting fillers in the PAAm matrix. However, creating effective conducting pathways requires mixing a high amount of the conducting filler (*36*, *38*), which can hinder the synthesis of a homogeneous hydrogel composite (*29*). The synthesis method for achieving a homogeneous hydrogel composite with high conductivity is illustrated in fig. S1. First, a less conductive PAAm hydrogel is synthesized by mixing a relatively low fraction of PEDOT:PSS into the PAAm medium (fig. S1, A and B). Then, the hydrogel composite is additionally functionalized with PANi to boost up the conductivity (fig. S1, C and D). In the PEDOT:PSS-PAAm hydrogel, the conductive elements, specifically the PEDOT:PSS particles, are dispersed within the poorly conductive PAAm hydrogel matrix (left inset of Fig. 2A). In contrast, the PEDOT:PSS-PANi-PAAm hydrogel facilitates rapid electron conduction by establishing three-dimensional (3D) conducting pathways within the PAAm hydrogel matrix (left inset of Fig. 2B). The 3D tomographic image shows the highly porous structure of the PEDOT:PSS-PAAm hydrogel (right inset of Fig. 2A) and the uniform distribution of PANi inside pores for the PEDOT:PSS-PANi-PAAm hydrogel (right inset of Fig. 2B). The detailed distribution can be observed (fig. S2, A and B). The content of PANi can also be estimated from the analysis (fig. S2C). The freeze-dried PEDOT:PSS-PAAm exhibits 43.8% voids, whereas PEDOT:PSS-PANi-PAAm shows 13.2% voids, suggesting that the PEDOT:PSS-PANi-PAAm hydrogel contains approximately 30.6% PANi.

As shown in Fig. 2C, despite the addition of a relatively low concentration (e.g., 5.5 wt %) of PEDOT:PSS to the PAAm hydrogel, the PEDOT:PSS-PAAm hydrogel composite starts to lose its homogeneity. Besides, as the weight fraction of PEDOT:PSS increases further to 10.8 wt %, the homogeneity deteriorates further and thereby the conductivity decreases down to 0.4 mS/cm. The homogeneous dispersion of PEDOT:PSS in the PAAm matrix can be maintained by 3.3 wt % PEDOT:PSS. Although a homogeneous hydrogel composite is obtained, its conductivity is only 20 mS/cm. This conductivity can be substantially boosted up through functionalization with PANi to ~24 S/cm (Fig. 2D).

To investigate the remarkably enhanced conductivity of the PEDOT:PSS-PAAm hydrogel upon functionalization by PANi, hydrogels were prepared by adjusting the aniline precursor concentration. Subsequently, the electrical conductivity of each hydrogel was measured and the results are shown in fig. S3A. At a low aniline concentration, the electrical conductivity shows a gradual increase. However, within the precursor ratio of aniline (ml) to acrylamide (g) ranging from 1 to 1.3, there is a substantial boost in electrical conductivity, reaching saturation at values less than 30 S/cm. Considering that PANi alone does not contribute to conductivity enhancement (fig. S3B), the substantial increase in electrical conductivity of PEDOT:PSS-PANi-PAAm hydrogel can be attributed to the interaction between the PEDOT:PSS and PANi molecules. Furthermore, despite cutting in various directions, the conductivity remained consistent (fig. S2, D to G). This observation suggests that the electrically conductive pathways formed through the interaction between PEDOT:PSS and PANi are uniformly distributed in a 3D manner.

The functionalization of the PEDOT:PSS-PAAm hydrogel with PANi also affects the impedance of the hydrogel. The impedance of the PEDOT:PSS-PAAm hydrogel is 2800 ohms at 1 kHz, and it is reduced to 21.7 ohms through PANi functionalization (Fig. 2D). The impedance spectra and phase angle over the full-range frequencies are presented in fig. S4. While the impedances of PAAm and PEDOT:PSS-PAAm hydrogels decrease with increasing frequency (fig. S4A), the impedance of the PEDOT:PSS-PANi-PAAm hydrogel remains consistently low irrespective of the frequency. This behavior is elucidated by examining the phase angle data, as shown in fig. S4B. The phase angle of the PEDOT:PSS-PANi-PAAm hydrogel is close to 0, indicating its dominance by resistance (*47*). Therefore, the PEDOT:PSS-PANi-PAAm hydrogel exhibits frequency-independent behavior in terms of impedance. To explore the influence of PANi functionalization on the impedance of the PEDOT:PSS-PANi-PAAm hydrogel, hydrogels were prepared by varying the aniline precursor concentrations. The impedance of each hydrogel was subsequently measured, and the results are shown in fig. S3C. At low aniline concentrations, the impedance of the hydrogel experiences a notable reduction. This trend continues as the precursor ratio of aniline (ml) to acrylamide (g) surpasses 0.5, eventually leading to the impedance level to approach saturation. The findings from the concentration adjustment experiment suggest that achieving low impedance requires PANi functionalization at low concentrations. In contrast, for reasonably high electrical conductivity, a substantially higher concentration of PANi functionalization is necessary.

The conduction mechanism of the PEDOT:PSS-PANi-PAAm hydrogel is also closely associated with molecular interactions, which can be explained in two main aspects: (i) The HCl component in the aniline solution (i.e., aniline, phytic acid, and 1 N HCl) leads to secondary doping of PEDOT:PSS; (ii) PANi molecules are synthesized along the doped PEDOT:PSS network. First, the secondary doping of PEDOT:PSS occurs through posttreatment using organic solvents or strong acids (*48*, *49*). As a result, phase separation and reorganization take place between PEDOT and PSS, causing a structural change in the benzoid state of the PEDOT chains to the quinoid state. This results in the formation of more accessible 3D fibrous networks rather than colloidal aggregation states. The same phenomenon can occur during the functionalization of PEDOT:PSS-PAAm hydrogel with PANi. X-ray photoelectron spectroscopy (XPS) analysis reveals that the functionalization of the PEDOT:PSS-PAAm hydrogel with the aniline solution results in a higher PEDOT/PSS ratio compared to the pristine PEDOT:PSS-PAAm hydrogel (fig. S5A). The result indicates the removal of the insulating PSS shells. In addition, the ultraviolet–visible–near infrared (UV-vis-NIR) absorbance result (fig. S5B) shows a decrease in absorbance in the polar band region (700 to 800 nm) and an increase in the bipolar band region (above 1100 nm). These results imply more efficient charge delocalization with a higher ratio of the quinoid state to the benzoid state (*50*). Second, PANi can undergo π-π interactions with PEDOT, and coulombic interactions can occur between the protonated NH group of PANi and the SO_3_^−^ group of PSS (*51*). As a result, the lamellar structures of PEDOT:PSS act as templates for PANi, enabling a plane attachment condition that promotes the homogeneous structure of the PEDOT:PSS-PANi-PAAm hydrogel. XPS analysis elucidates the complementary doping effect of PSS on PANi. In the case of PEDOT:PSS-PAAm hydrogel (fig. S5C), the peaks at 397.7, 399.1, and 401.3 eV in the N1s spectra correspond to nitrogen in the cross-linking agent [*N,N*-methylene bis-acrylamide (MBAA)], the amide group in PAAm, and the nitrogen of some quaternary amide formed during hydrogel synthesis, respectively (*52*). However, for PEDOT:PSS-PANi-PAAm hydrogel (fig. S5D), the spectra show five main chemical states, including the amide group in PAAm at 399.1 eV, quinoid imine at 399.8 eV (═N─), the benzenoid imine at 400.6 eV (─NH─), the protonated amine at 401.5 eV (─NH_2_^+^), and protonated imine (═NH^+^) at 402.1 eV. The complementary doping of the PANi chain with sulfonic acid in PSS is attributed to the generation of positively charged radical nitrogen (*53*). Therefore, the conformation transformation of PEDOT:PSS and strong interaction between PEDOT:PSS and PANi contribute synergistically, resulting in high conductivity.

Another key material characteristic is the pH dependency. Conducting polymers such as PEDOT:PSS (*54*) and PANi (*55*) show pH-dependent conductivity changes. Their conductivity increases because of doping with H^+^ under acidic conditions, while the conductivity rapidly decreases as pH increases. This pH dependency is useful for pH sensing in various organs (skin: ~5, gastric fluid: ~2, and others: ~7) (*56*, *57*). Note that the conductivity increase by the PANi doping is effective in both acidic and basic conditions (240 to 666 times conductivity increase over the pH range of 1 to 7; Fig. 2E). In conjunction, the impedance of the hydrogel is also influenced by pH. The impedance of the PEDOT:PSS-PANi-PAAm hydrogel is 23.7 ohms at pH 1 (Fig. 2E). It slightly increases with raising the pH of environments and reaches 157 ohms at pH 7. In comparison, although the PEDOT:PSS-PAAm hydrogel exhibits a low impedance of 37 ohms at pH 1, the impedance is markedly increased at higher pH. In addition, the PEDOT:PSS-PAAm hydrogel shows frequency dependency, with a notable increase in impedance at lower frequencies (fig. S6A). For instance, the hydrogel has an impedance of over 1 kilohm at 10 Hz. In contrast, the PEDOT:PSS-PANi-PAAm hydrogel maintains a consistent impedance level irrespective of the frequency (fig. S6B).

To evaluate the mechanical properties of the hydrogels including stretchability and softness, stress-strain curves were measured (Fig. 2F). The high stretchability of the PAAm hydrogel decreases as the functionalization proceeds. However, the PEDOT:PSS-PANi-PAAm hydrogel still shows a high stretchability of 358%, which exceeds the elongation limit of various tissues and organs (Fig. 1J). While stretching from 0 to 300%, the electrical conductivity of the hydrogel was also measured (fig. S7A). Throughout this stretching range, the initial resistance of the PEDOT:PSS-PANi-PAAm hydrogel increased by approximately 39% (fig. S7B). The Young’s modulus of PEDOT:PSS-PANi-PAAm is calculated as ~14 kPa, which is much lower than that of skin (50 to 150 kPa) (*58*) and comparable to that of heart (10 to 15 kPa) (Fig. 1I). PEDOT:PSS-PANi-PAAm exhibits highly stretchable and soft nature, making it well suited for bioelectronic applications from a mechanical perspective and alleviating mechanical mismatch between bioelectronics and target tissues.

For the wearable device applications, the dehydration issue of hydrogels could be a critical issue. We evaluated the mechanical and electrical properties of the hydrogel by adjusting the degree of hydration through drying (fig. S8). As drying progresses, a reduction in water content is observed, leading to an increase in the modulus and stretchability of the hydrogel. In addition, there is a tendency of an increase in electrical conductivity. However, since the further dehydration can substantially alter the performance of the hydrogel, it is necessary to prevent this situation by covering the hydrogel with an appropriate encapsulation layer (*33*).

### Strategies for improving adhesive properties of the conductive hydrogel

One of the most widely used methods for integrating hydrogels with soft electronics is the “cut-and-place” method (*33*). However, during the fabrication and application of the device, excess water can result in loss of contact between the hydrogel and the electrode even with small sheer force (fig. S9A). In the 90° peeling test, hydrogels placed on the skin (porcine skin) or metal substrate using the cut-and-place method exhibit very low adhesion characteristics (fig. S10). This issue can be solved by using on-device hydrogel polymerization, interfacial covalent bonding, and efficient energy dissipation strategies (fig. S9B).

**Fig. 3.**
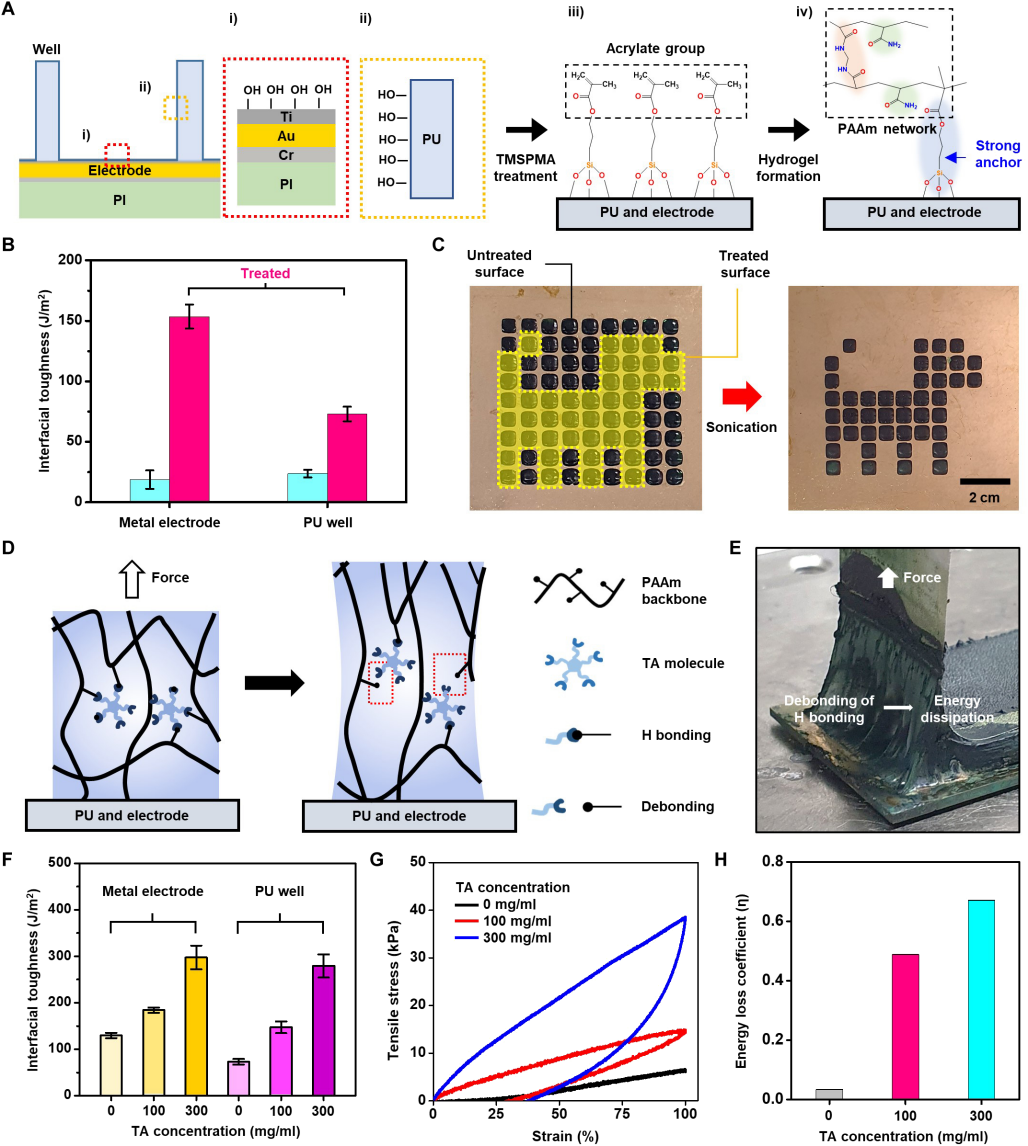
Strong adhesion between conductive hydrogels and electrodes/stretchable wells. (**A**) Schematic illustration describing the chemical integration of hydrogels on the electronic device. (**B**) Adhesion properties of the conductive hydrogel on untreated and chemically treated metal electrodes and PU wells. (**C**) Sonication of the conductive hydrogels formed on untreated and chemically treated electrode surfaces. (**D**) Schematic illustrations to show the concept of the adhesion enhancement through energy dissipation. (**E**) Image of the PEDOT:PSS-PANi-PAAm hydrogel undergoing a peeling test. (**F** and **G**) Improved adhesion (F) and loading-unloading test results (G) before and after the TA treatment of the PEDOT:PSS-PANi-PAAm hydrogel. (**H**) Energy loss coefficient calculated from the loading-unloading test results.

As shown in Fig. 3A, the electrode, on which the hydrogel is polymerized, consists of three thin-film layers. Cr (10 nm) is used as a binding layer to the polyimide (PI) (2 μm) substrate. Au is used as a signal transporting layer (100 nm). Ti (10 nm) is added for hydrogel attachment. Ti surface can be easily oxidized as well as resistant to strong acid environments (fig. S11), allowing for stable formation of functional groups on its surface over a wide range of pH (Fig. 3A, i). The surface of the PU well can also be oxidized for functionalization (Fig. 3A, ii). Next, a self-assembled monolayer, 3-(trimethoxysilyl) propyl methacrylate (TMSPMA), is coated on the oxidized surfaces of Ti and PU, forming acrylate functional groups (Fig. 3A, iii). The hydrogel precursor is polymerized in the PU well in situ by using UV light, and then the hydrogel can be firmly adhered to both surfaces (Fig. 3A, iv).

To test the adhesion property of the hydrogel, 90° peeling test was performed (Fig. 3B and fig. S12, A and B). PEDOT-PSS:PANi-PAAm exhibits the interfacial toughness of 17.3 and 23.7 J/m^2^ on the bare electrode and PU well (i.e., nonfunctionalized surface), respectively. These values can be increased to 153.6 and 72.9 J/m^2^ on the functionalized electrode and PU well due to the chemical anchors. Note that the typical fracture energy of pure single network hydrogels (*59*) is <100 J/m^2^. Another challenge arises from the fact that the presence of large amounts of conducting polymers usually hinders adhesion to the substrate. As shown in figs. S13A and S14 (A and B), we successfully synthesized a PAAm hydrogel with robust bonding capability by optimizing the ratio of acrylamide to MBAA. The interfacial toughness of the PAAm hydrogel on the functionalized electrode reaches 421 J/m^2^. However, in the case of the PEDOT:PSS-PANi-PAAm hydrogel, the interfacial toughness decreases to 153 J/m^2^. This highlights the difficulty of achieving both conductivity and strong adhesion simultaneously. However, our functionalized hydrogel exhibits notably high adhesion properties due to the adhesion layer design and surface functionalization. These hydrogels maintain the adhesion well even under sonication (over 10 min @ 40% amplitude) (e.g., Fig. 3C; good adhesion is shown only on the chemically treated region). The adhesive characteristic is well maintained not only under mechanical peeling or sonication but also under acidic conditions. As shown in figs. S13B and S14 (C and D), the interfacial toughness of the PEDOT:PSS-PANi-PAAm hydrogel remains stable even when immersed in a 1 N HCl solution for 6 hours. On the contrary, while pure PAAm hydrogel initially exhibits high interfacial toughness of 421 J/m^2^, it decreases considerably over time. After 6 hours of immersion, its interfacial toughness is lower than that of the PEDOT:PSS-PANi-PAAm hydrogel.

To improve the adhesion further, the hydrogel is treated by a hydrogen bond–forming agent [tannic acid (TA)], leading to enhanced energy dissipation and adhesion under mechanical deformation (Fig. 3D) (*60*). When the TA-treated hydrogel is detached from the substrate (chemically treated metal electrode), the hydrogel is stretched without breaking (Fig. 3E). The toughness at the hydrogel-electrode and the hydrogel-PU well is markedly improved from 129.6 to 297.6 J/m^2^ and from 72.9 to 279.2 J/m^2^ by the TA treatment, respectively (Fig. 3F and fig. S12, C and D), which are comparable to those obtained when using catechol (*61*). These enhancements can be explained by the energy dissipation observed in the loading-unloading test (Fig. 3G). The hysteresis in the stress-strain curve indicates that the hydrogel undergoes energy dissipation during deformation. In addition, the energy loss coefficient is notably increased with the TA treatment (Fig. 3H). This phenomenon can be due to hydrogen bonds formed between the hydrogel polymer and TA (fig. S15).

### Fabrication of stretchable bioelectronics integrated with conductive hydrogels

**Fig. 4.**
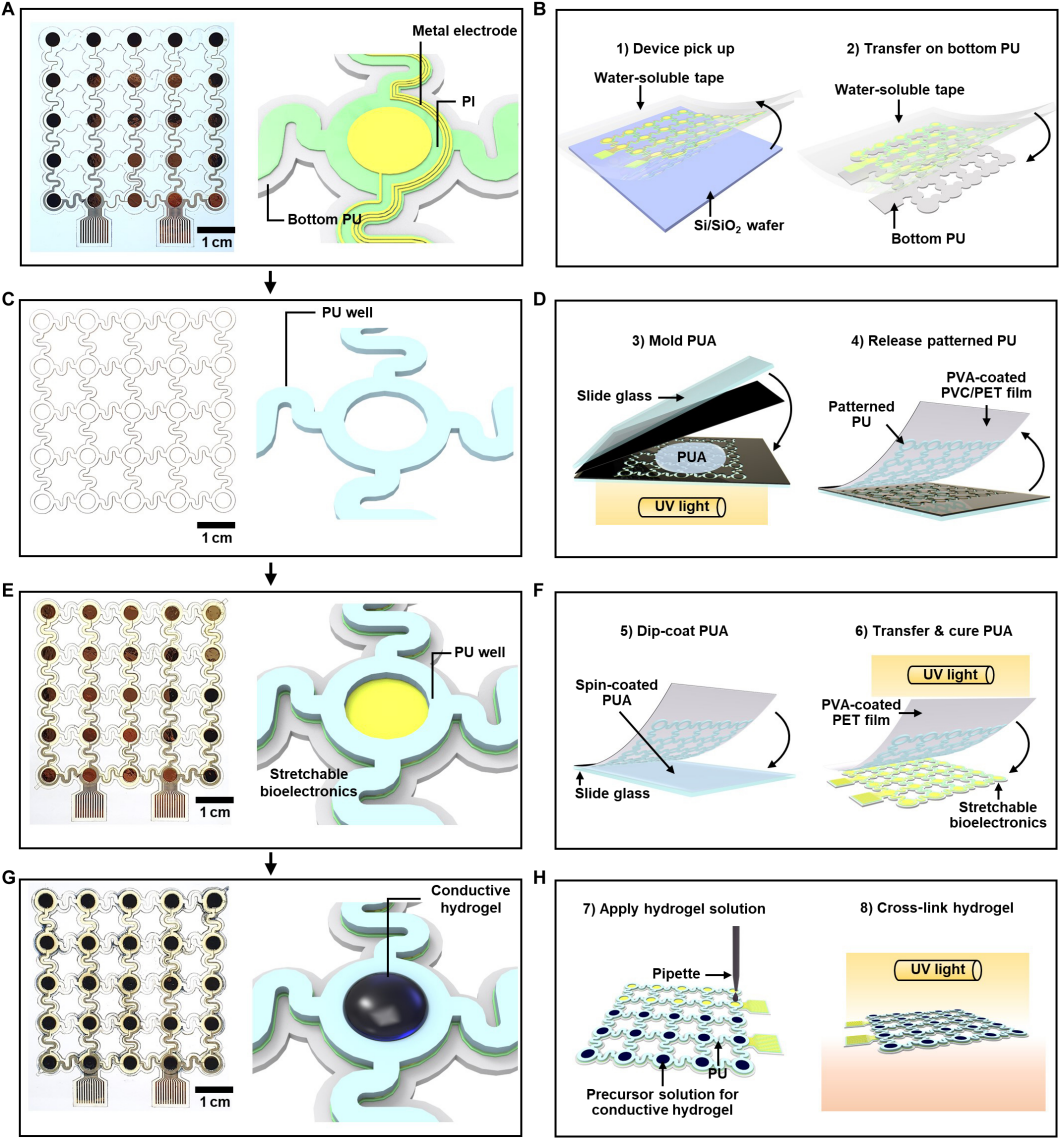
Fabrication process of the stretchable bioelectronics integrated with the conductive hydrogel. (**A**) Photograph (left) and schematic illustration (right) of the stretchable bioelectronics (before integration of the conductive hydrogel). (**B**) Pick-up and transfer process of the stretchable device. (**C**) Photograph (left) and schematic illustration (right) of the elastic PU well. (**D**) Fabrication of the PU well and its transfer printing to the PVA-coated film. (**E**) Photograph (left) and schematic illustration (right) of the integrated device. (**F**) Integration process using the PUA glue. (**G**) Photograph (left) and schematic illustration (right) of the stretchable bioelectronics with the conductive hydrogel. (**H**) In situ formation of conductive hydrogels inside the PU wells.

Figure 4 depicts fabrication steps of the stretchable bioelectronics integrated with the conductive hydrogel. First, the stretchable bioelectronics is fabricated (Fig. 4A and fig. S16A). A PI layer is coated on a Si/SiO_2_ wafer, and then the circular-shape and serpentine-shape stretchable electrodes (Cr/Au/Ti) are fabricated on the surface of the PI layer. After encapsulation of the electrode with epoxy, it is transferred onto a prepatterned PU substrate (Fig. 4B). To fabricate the elastic PU wells (Fig. 4C and fig. S16B), a mold is prepared by using a laser cutting technique, with plastic films on the sidewalls and glass as a bottom substrate. A prepolymer solution containing of PU acrylate (PUA) is applied to the mold, which is then covered with a polyvinyl alcohol (PVA)–coated polyethylene terephthalate (PET) film (Fig. 4D, left). By selectively exposing PUA to UV light, the elastic PU wells are fabricated. When the cover is peeled off from the mold, a stretchable pattern of the PU wells adheres to the surface of the PVA-coated cover due to the sticky PVA coating layer (Fig. 4D, right). Then, the stretchable bioelectronics is integrated with the PU wells (Fig. 4E). The bottom surface of the PU well is coated with precured PUA that serves as a glue, and the PU well array is carefully aligned with the stretchable bioelectronics (Fig. 4F, left). The PUA glue subsequently is cured by UV exposure, forming robust bonds between the device and the PU well (Fig. 4F, right).

Next, the conductive hydrogel is formed on the electrode inside the PU well in situ and functionalized to enhance its electrical and adhesive properties (Fig. 4G). Before the in situ formation of the conductive hydrogel, the surfaces of the metal electrode and PU well are treated with oxygen plasma reactive ion etching (O_2_ plasma RIE) to form oxidized surfaces. Subsequently, a self-assembled monolayer of TMSPMA is deposited on the treated surfaces. To synthesize the hydrogel, a PAAm precursor solution containing PEDOT:PSS is applied inside the PU well, and a PEDOT:PSS-PAAm hydrogel is polymerized by UV exposure (Fig. 4H). During the polymerization process, the acrylate group of TMSPMA is simultaneously cross-linked with the network of the PAAm hydrogel, establishing the strong covalent bond between the hydrogel and the stretchable device. Last, the PEDOT:PSS-PAAm hydrogel is functionalized with PANi and TA for enhancing the electrical and adhesion properties.

### Stretchable multichannel sensor array for in vitro and in vivo measurement of impedance and pH

**Fig. 5.**
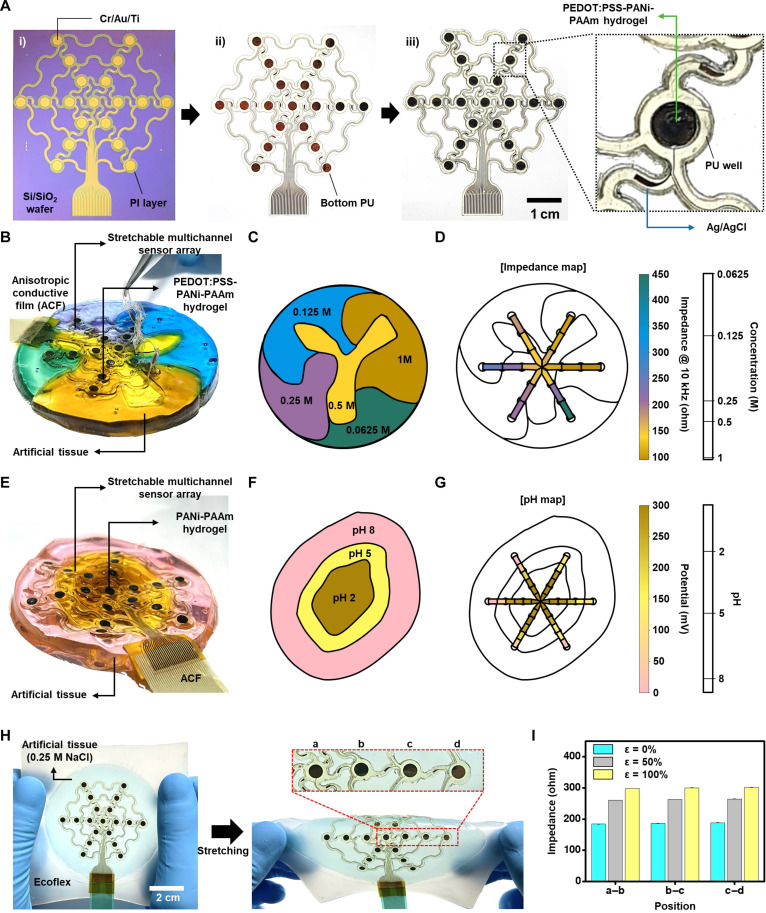
Fabrication of stretchable multichannel sensor array and in vivo impedance and pH mapping demonstration. (**A**) Fabrication steps for the stretchable multichannel sensor array. (**B**) Stretchable multichannel sensor array on the artificial tissue with various ion concentrations. (**C**) Spatial variation of ion concentrations in the artificial tissue. (**D**) Impedance mapping on the artificial tissue. (**E**) Stretchable multichannel sensor array on the artificial tissue with various pHs. (**F**) Spatial variation of pHs in the artificial tissue. (**G**) pH mapping on the artificial tissue. (**H**) Images of the stretchable multichannel sensor array on the artificial tissue before (left) and after stretching (right). (**I**) Impedance mapping from each position during stretching deformations.

To verify the performance of the stretchable bioelectronics integrated with the homogeneously conductive hydrogels and the effect of the seamless low-impedance tissue-device interface, we demonstrate the in vitro and in vivo measurement of impedance and pH with the multichannel sensor array (Fig. 5A). The unit cell (right inset of Fig. 5A) consists of the conductive hydrogel (PEDOT:PSS-PANi-PAAm hydrogel for impedance measurement and PANi-PAAm hydrogel for pH measurement) and Ag/AgCl electrodes formed on Cr/Au/Ti electrodes, which are connected to serpentine-shape stretchable interconnections. Detailed fabrication procedures follow the process explained in Fig. 4.

To demonstrate impedance and pH measurement in vitro, two types of artificial tissue are prepared. The artificial tissues are prepared with PAAm-alginate hydrogel and have different ion concentrations and pH levels (fig. S17). The stretchable multichannel bioelectronics is attached on the artificial tissues with different ion concentrations (Fig. 5B). The device measures the impedance between adjacent electrodes containing PEDOT:PSS-PANi-PAAm hydrogel (Fig. 5, C and D, and fig. S18). The PANi-doped PAAm hydrogel working electrode is used with an Ag/AgCl reference electrode (Fig. 5E) (*62*) for the pH measurement (Fig. 5, F and G, and fig. S19). PANi exhibits conductivity changes corresponding to pH variations. Thus, pH can be determined by measuring the difference in electromotive force between the PANi-doped PAAm hydrogel electrode and the Ag/AgCl electrode. The pH sensor displays a pH sensitivity of −33.94 mV/pH (fig. S19, A and B). In addition to mapping under static conditions, the stretchable design of the device, along with the mechanical and electrical properties of the conductive hydrogel, enables the dynamic measurement of signals. To demonstrate this characteristic of the stretchable multichannel sensor array, measurement under dynamic condition was performed. For this demonstration, the device was placed on an artificial tissue containing 0.25 M NaCl, subjected to stretching up to 100% (Fig. 5H, fig. S20A, and movie S1), and impedance was concurrently measured at multiple points (Fig. 5I). A layer of Ecoflex was used as a handling substrate to stretch both the device and the artificial tissue. At each position, similar levels of impedance were measured, and an increase in impedance was observed by stretching (Fig. 5I). The results of the stretching cycle test indicate a restoration of the initial impedance levels when the applied strain becomes zero (fig. S20, B to D), affirming the potential of the stretchable multichannel sensor array as a promising tool for biomedical applications, even under stretchable conditions.

Before in vivo bioelectronic applications, we evaluate biocompatibility of the conductive hydrogel through cell viability and proliferation tests. Specifically, C2C12 cells were incubated with a hydrogel extract solution for up to 72 hours, and their viability and proliferation state were assessed. The cell proliferation tests show no notable difference between the experimental groups incubated in five different hydrogel extract solutions and the control group incubated in a growth medium (fig. S21A). Furthermore, the cell viability remains consistently high, with almost 100% viability observed in all cases (fig. S21B). These results confirm the biocompatibility of the conductive hydrogels synthesized in this study.

Last, we demonstrate the in vivo impedance and pH measurement of the rat skin and rat gastric fluid. For the impedance measurement, a skin burn wound model is prepared (fig. S22A). The rat skin is contacted with a heated copper cylinder, which causes a superficial skin injury without blister formation (*63*). Because of the skin burn, the skin impedance increases (fig. S22B). Then, the second skin burn is made, which leads to a higher skin impedance than that of the first burn, which can be attributed to the skin tissue structure change as well as the skin moisture loss. For the in vivo pH measurement, the gastric wall of a rat is incised and the pH sensor (PANi-PAAm hydrogel on Cr/Au/Ti electrode and Ag/AgCl on Cr/Au electrode) is placed inside a rat stomach (fig. S22C). When the device contacts the gastrointestinal fluid, a potential of 229.5 mV is measured (fig. S22D), which corresponds to the potential of a pH 2 buffer solution (fig. S19B).

## DISCUSSION

We have presented a series of material and interface control strategies for the stretchable bioelectronics by overcoming existing challenges in electrical performance, material homogeneity, and monolithic device integration. One strategy involves creating a homogeneously conductive hydrogel with an exceptionally low impedance and a reasonably high conductivity by synthesizing a homogeneous PAAm hydrogel with low-concentration PEDOT:PSS and decorating it with PANi. Another important strategy is the monolithic firm integration of the conductive hydrogel with the stretchable bioelectronics by using on-device polymerization as well as incorporating surface covalent bonding and bulk hydrogen bonding. These strategies enable the fabrication of the stretchable multichannel sensor array for impedance and pH measurement in vitro and in vivo. These material and interfacial control strategies open opportunities for the high-performance soft bioelectronics.

## MATERIALS AND METHODS

### Fabrication of the patterned PU elastomer including bottom layer and well pattern

To prepare a mold for the patterned PU elastomer, three sheets of 100-μm-thick PET film with an adhesive layer were stacked on a glass substrate. Commercially available polyvinyl chloride (PVC) and PI tapes were subsequently attached onto the PET surface. The PVC tape serves as a mask, preventing light from penetrating during the UV polymerization of PUA, while the PI tape prevents thermal degradation during laser cutting, improving the resolution of the mask. The attached films were cut using a laser cutting machine (VLS 2.30, Universal Laser Systems) and washed with ethanol and deionized (DI) water. To prepare the cover of the mold, a PVC tape was attached on a PET film and treated with O_2_ plasma RIE [100 standard cubic centimeters per minute (sccm), 0.1 torr, 100 W, 30 s]. After treatment, a 20 wt % aqueous PVA solution prepared with PVA (363170, Sigma-Aldrich) was spin-coated (1000 rpm for 30 s) and heated at 95°C for 1 min. Before the molding process, the surface was treated with O_2_ plasma RIE (100 sccm, 0.1 torr, 150 W, 30 s). Subsequently, a mold release agent (HYUNDAI mould releases special, soybean oil spray type) was sprayed onto the mold and dried on a hot plate at 95°C for 1 min. Next, PUA (MCNet, SPU-8000) was applied to the mold and covered with the PVA-coated PET cover. After curing the PUA in a UV curing machine (Fusion Cure System, Minuta Technology) for 7 min, the cover was peeled from the mold and the UV-cured PU layer was attached to the PVA layer. Last, the sample was washed with acetone, ethanol, and DI water.

### Fabrication of the stretchable multichannel electronic device

A PI layer was formed on a wafer by spin coating poly(pyromellitic dianhydride-co-4-4′-oxydianiline), amic acid solution (575798, Sigma-Aldrich) at 2000 rpm for 60 s, and subsequent thermal imidization. Next, a layer of negative photoresist (AZ nLOF 2070, MicroChemicals GmbH) was patterned on the PI surface, and Cr/Au/Ti layers (10/100/10 nm) were deposited through thermal evaporation. Using a lift-off process in an acetone bath, the electrode pattern of the stretchable multichannel electronic device was defined on the PI layer, followed by encapsulating the electrode pattern with a layer of epoxy (SU-8 2, Kayaku Advanced Materials). The PI layer was then patterned using a positive photoresist (AZ P4620, MicroChemicals, GmbH) and treated with O_2_ plasma RIE. To transfer the electrode pattern on the PI layer onto a PU substrate, a water-soluble tape (5414, 3M) was attached and gently peeled. The backside (PI surface) of the water-soluble tape and the patterned bottom PU layer were treated with O_2_ plasma RIE (100 sccm, 0.1 torr, 100 W, 30 s). The bottom PU layer was pressed onto the spin-coated layer of UV-curing optical adhesive (Norland Optical Adhesive, NOA 73). The water-soluble tape was attached to the PU layer and fixed with forceps, cured for 7 min, and removed with DI water. After soaking in DI water for 10 min, the PVA layer was peeled off. The top PU layer was transferred in a similar manner. The substrate and the top PU layer were treated with O_2_ plasma RIE (100 sccm, 0.1 torr, 100 W, 30 s). The top PU layer was pressed onto the spin-coated layer of optical adhesive, fixed on the substrate, and cured for 7 min. After the PVA layer was removed with DI water, the stretchable electronic device was obtained. To enable pH measurement, Ag/AgCl electrodes were also fabricated. The electrode was prepared by applying Ag/AgCl ink (011464, ALS) to the Cr/Au/Ti electrode and drying it on a hot plate at 125°C for 3 min.

### Synthesis and integration of the highly conductive hydrogels onto the stretchable device

To create the covalent bonding of the conductive hydrogels with the PU wells and the bottom electrodes, the device surface was treated with O_2_ plasma RIE (100 sccm, 0.2 torr, 30 W, 5 min) and TMSPMA. A solution for the TMSPMA treatment was prepared by adding 2 ml of TMSPMA (M6514, Sigma-Aldrich) to 100 ml of DI water containing 10 μl of acetic acid. The device was then treated for 2 hours and washed with ethanol and DI water. To prepare PEDOT:PSS-PANi-PAAm hydrogels, PEDOT:PSS (Clevious PH1000, Heraeus Electric Materials) was filtered with a 0.8-μm cellulose acetate membrane filter and freeze-dried. The freeze-dried PEDOT:PSS (0.03 g) was dissolved in 5.33 ml of DI water by stirring for 1 hour. Subsequently, 0.66 ml of ethylene glycol was added and stirred for 30 min. Next, 0.904 g of acrylamide (A8887, Sigma-Aldrich) and 0.0034 g of MBAA (146072, Sigma-Aldrich) were added and degassed. Then, 50 μl of 10 wt % ammonium persulfate (APS; A3678, Sigma-Aldrich) and 4.5 μl of *N,N,N′,N′-*tetramethyl ethylenediamine (TEMED; T9281, Sigma-Aldrich) were added. The solution was dropped over the mold, covered with a Teflon-coated glass (Teflon AF 1600, DuPont), and cured under UV light for 90 min. To further enhance electrical conductivity, the hydrogel was functionalized with PANi. The hydrogel was immersed in a mixture containing 45 ml of 1 N HCl solution (000H0423, Samchun Chemicals), 6.6 ml of phytic acid (593648, Sigma-Aldrich), and 1.16 ml of aniline (242284, Sigma-Aldrich) and shaken for 2 hours. Next, 0.285 g of APS was added, shaken for 4 hours, and then washed with DI water. To create hydrogen bonding within the hydrogel, the hydrogels were treated in a TA (403040, Sigma-Aldrich)–containing bath (100 and 300 mg/ml) for 6 hours. To prepare PANi-PAAm hydrogels, PAAm hydrogels were prepared. First, a precursor solution was prepared by adding 1.56 g of acrylamide, 70 μl of acrylamide/bis-acrylamide 30% solution (A3449, Sigma-Aldrich), 50 μl of 10 wt % APS, and 7 μl of TEMED to 10 ml of DI water. The solution was then dropped and cured under UV light for 90 min. The functionalization with PANi was carried out in the same manner as mentioned above.

### Characterization of microstructure, as well as electrical, mechanical, and adhesion properties of hydrogels

Two hydrogel samples were prepared for 3D tomography: The first hydrogel was prepared by incorporating a concentration of 3.3 wt % PEDOT:PSS to a PAAm hydrogel (PEDOT:PSS-PAAm hydrogel). The second sample was prepared by decorating the PEDOT:PSS-PAAm hydrogel with PANi, resulting in the PEDOT:PSS-PANi-PAAm hydrogel. The hydrogel samples were freeze-dried and scanned using a ZEISS Xradia 620 Versa x-ray microscope (XRM; Carl Zeiss). Conductivity measurement of hydrogel films (40 mm by 10 mm by 100 μm) was performed using a four-point probe and connected to a source meter (2450, Keithley). To measure the electrical conductivity of hydrogels according to pH, the hydrogels were soaked in pH buffer solutions for 1 day before the measurement. To assess homogeneity in the electrical conductivity of the hydrogels, two experiments were conducted. First, a 25 mm–by–15 mm–by–100 μm hydrogel was longitudinally divided into 10 sections to measure conductivity. Second, a 7 mm–by–15 mm–by–100 μm hydrogel was soaked in an embedding medium (O. C. T. compound, Tissue-Tek, Sakura) for 1 day, then frozen for 1 day, and divided into 10 sections in the thickness direction using a cryotome (CM1850, Leica) for conductivity measurement. For impedance measurement, two Pt electrodes (1 cm by 5 cm) were deposited on a wafer with a distance of 3 mm and encapsulated, leaving 1 cm–by–1 cm area between them. A hydrogel film was placed to cover both electrodes and then dried in an oven at 60°C for 3 min. Impedance was measured using an electrochemical analyzer (CHI660E, CH Instruments) with a frequency range of 10^0^ to 10^5^ Hz. To measure the impedance of the hydrogels according to pH, the hydrogels were soaked in pH buffer solutions for 1 day before the measurement. For mechanical characterization, samples were prepared in a dogbone shape and tested using a mechanical testing machine (34SC-1, Instron) at a rate of 10 mm/min. To assess the mechanical and electrical properties of the hydrogel with varying hydration degrees, a natural convection oven (C-DoD1, Chang Shin CO.) was used for drying at 80°C. Swollen hydrogels were subjected to drying intervals of 0, 7.5, 15, 22.5, and 30 min. Then, modulus, stretchability, and conductivity of the hydrogels were measured. For adhesion measurement, layers of Cr/Au/Ti (10/100/10 nm) were deposited on a slide glass (2.5 cm by 7.5 cm) by thermal evaporation, which were then treated with TMSPMA. A PET frame (2 mm thick) was attached, and a hydrogel monomer solution was dropped inside the frame. Stainless steel gauze (type 316, Goodfellow) was used as a backing layer for the hydrogel and treated with TMSPMA before being attached to a PET film to serve as a cover film. The stainless-steel gauze was slightly longer than the glass, allowing it to be secured to the crosshead of a universal testing machine (ESM301, Mark-10). The bottom of the glass was attached to a 90° peeling fixture (G1045, Mark-10) using double-sided tape (VHB, 3M), and peeling was conducted at a rate of 50 mm/min.

### XPS and UV-vis-NIR spectra of conductive hydrogels

XPS analysis (AXIS SUPRA) was used to examine the PEDOT:PSS ratio and its chemical states before and after treatment with an aniline solution. PEDOT:PSS-PAAm hydrogel samples were prepared, freeze-dried, and pressed into pellet forms using a manual hydraulic press (Atlas 15T) with a force of 10 tons. S2p spectra were analyzed with a monochromated Al Kα source. To investigate the interaction between PEDOT:PSS and PANi, PEDOT:PSS-PAAm and PEDOT:PSS-PANi-PAAm hydrogels were prepared into pellet forms, and N1s spectra were analyzed. UV-vis-NIR spectroscopy (V-770, JASCO) was used to assess the doping level of PEDOT chains before and after treatment with an aniline solution. The absorbance of fully dried hydrogel films was measured from 300 to 1800 nm.

### Reactivity of metals in acidic condition

To assess the reactivity of metals in an acidic environment, disk patterns (diameter: 20 μm) of Cr, Au, and Ti were formed on Si/SiO_2_ wafers. Wet etching methods were used to pattern Al and Cr. Metal films of 100 nm were deposited through thermal evaporation, followed by patterning of positive photoresist (MIROPOSIT S1805, DOW). Etching was performed using Al etchant (type D, TRANSENE) and Cr etchant (CE-905N, TRANSENE). For Ti patterning, negative photoresist (AZ nLOF 2070, MicroChemicals, GmbH) was used, followed by a thermal deposition of 100 nm on the wafer. Subsequently, a lift-off process was conducted in an acetone bath to define the pattern. Before the characterization, surface cleaning was conducted to remove any organic residues on the samples by treating with O_2_ plasma RIE (100 sccm, 0.1 torr, 100 W, 30 s). The surface roughness profile of the as–surface cleaned metal thin films was obtained using an atomic force microscope (NX-10, Park Systems). To examine the reactivity of metals in acid, the metals were immersed in a 1 N HCl bath for 6 hours, and their surface roughness profiles were obtained.

### Preparation of the artificial tissue

To prepare a hydrogel with rough surface, simulating the texture of human tissue, a thin layer of epoxy (SU-8 100, Kayaku Advanced Materials) was applied to a glass substrate. After 2 min of UV exposure, it was cured on a hot plate at 150°C for 30 min. Then, a PET spacer (2 mm thick) was attached to the edge of the epoxy layer, and PAAm-alginate hydrogel was formed by adding 2.028 g of acrylamide, 0.2 g of sodium alginate (W201502, Sigma-Aldrich), 91 μl of acrylamide/bis-acrylamide 30% solution, 50 μl of 10 wt % APS solution, and 7 μl of TEMED to 10 ml of DI water, and curing for 2 hours. The synthesized hydrogel was patterned using a laser cutting machine, and color dyes (Chefmaster Liqua-gel) were used to add color to the hydrogel pieces.

### Impedance and pH mapping

For impedance mapping, the hydrogel pieces were immersed in sodium chloride (S9888, Sigma-Aldrich) solutions with specific colors: 1 M (orange), 0.5 M (gold), 0.25 M (purple), 0.125 M (blue), and 0.0625 M (green). For calibration, each piece was mounted on two sets of PEDOT:PSS-PANi-PAAm hydrogel with a diameter of 3 mm robustly bound on a Cr/Au/Ti electrode. After each measurement, the hydrogels were washed with DI water and air-dried for 1 min. Impedance was measured in the frequency range of 10^3^ to 10^5^ Hz. The stretchable multichannel electronic device was placed on top of the assembled hydrogel pieces, and impedance between the hydrogels was measured. For pH mapping, the hydrogel pieces were immersed in pH buffer solutions with specific colors: pH 2 (brown), pH 5 (gold), and pH 8 (pink). For calibration, each piece was mounted onto a pair of a PANi-PAAm hydrogel formed on a Cr/Au/Ti electrode and Ag/AgCl ink coated on a Cr/Au electrode. The open circuit potential between the electrodes was measured using an electrochemical analyzer. After each measurement, the hydrogels were carefully washed with Dulbecco’s phosphate-buffered saline (DPBS; D8537, Sigma-Aldrich) for 2 min and dried in an oven for 3 min. The stretchable multichannel electronic device was positioned on top of the assembled hydrogel pieces, and the open circuit potential was measured between the hydrogels and Ag/AgCl electrodes.

### Impedance measurement under dynamic condition

Impedance measurements were conducted under dynamic condition using the stretchable multichannel sensor array. A layer of Ecoflex (00-50, Smooth-On) served as the stretchable substrate, prepared in dimensions of 10 cm by 10 cm by 1 mm. A 10 wt % benzophenone solution (B9300, Sigma-Aldrich) in ethanol was applied for 3 min, followed by washing the surface with ethanol and DI water to achieve robust bonding with the hydrogel. An artificial tissue model using PAAm-alginate hydrogel was then synthesized on the Ecoflex substrate, measuring 7 cm in diameter and containing 0.25 M sodium chloride. Subsequently, the stretchable multichannel sensor array was applied and subjected to stretching. The Ecoflex substrate was horizontally stretched, and impedance between the two electrodes was measured at 50 and 100% strain levels.

### Animal-related experiments

Our animal experiments complied with the Korea Food and Drug Administration guidelines, and the procedures were approved by the Seoul National University Institutional Animal Care and Use Committee (permission number: SNU-210330-2). Eight-week-old male rats (Orient Bio Inc.) were anesthetized with intraperitoneal injection of urethane (Merck) solution (1.4 g/kg). After deep anesthesia was confirmed with toe pinch, the dorsal hair was removed with commercial hair removal cream. Then, the naked skin was washed with DI water. To create a model of skin burns, a copper cylinder with a diameter of 36 mm was placed on a hot plate at 150°C for 10 min. For primary skin burn, the copper cylinder was gently pressed onto the skin for 10 s, and for secondary burn, it was pressed for an additional 10 s. Skin impedance was measured using two sets of PEDOT:PSS-PANi-PAAm hydrogel formed on a Cr/Au/Ti electrode. For pH measurement with gastrointestinal fluid, abdominal skin was disinfected with povidone. The abdominal skin and muscle were incised for 2 cm with iris scissors. Then, the gastric wall was incised for 1 cm with sterilized iris scissors. A pair of a PANi-PAAm hydrogel on Cr/Au/Ti electrode and an Ag/AgCl ink on Cr/Au electrode was inserted through the incision to contact the gastrointestinal fluid.

### Cell viability assay

The cytotoxicity of hydrogels was evaluated according to the ISO 10993-12 standard. To prepare a hydrogel extract solution, 0.03 g of hydrogel was immersed in 10 ml of Dulbecco’s modified Eagle’s medium (Gibco) supplemented with 10% fetal bovine serum (Gibco) and 1% penicillin-streptomycin (Gibco) (growth medium) at 37°C in a humidified atmosphere of 5% (v/v) CO_2_ for 24 hours. The solutions were then filtered with a 0.2-μm syringe filter (Pall Corporation) for sterilization. The mouse myoblast C2C12 cell line was purchased from the American Type Culture Collection (ATCC; CRL-1772). Cells were cultured in growth medium at 37°C in a humidified atmosphere of 5% (v/v) CO_2_. At a confluence of 80 to 90%, the cells were treated with 0.05% trypsin-EDTA (Gibco) and seeded on a 96-well plate with a density of 1.5 × 10^3^ cells/cm^2^ and cultured with the growth medium. After 4 hours of incubation, the growth medium was replaced with the hydrogel extract solution. A control group was also evaluated by incubating cells with the growth medium. Cell viability was evaluated using a live/dead staining kit (Biomax). After 24, 48, and 72 hours of incubation with the hydrogel extract solution, the cells were incubated with 2 μM calcein AM and 3 μM propidium iodide in 5 ml of DPBS for 1 hour at 37°C. The stained samples were observed using a fluorescence microscope (Ti-S/L100, Nikon). Cell proliferation was determined using the Cell Counting Kit-8 (CCK-8, Dojindo). After 24, 48, and 72 hours of incubation with the hydrogel extract solution, the reagents were added to each well and incubated for 3 hours at 37°C according to the manufacturer’s protocol. Absorbance at 450 nm was measured by a microplate reader (SpectraMax M3, Molecular Devices). Cell proliferation was evaluated as a percentage value relative to the control group.
