# Supplementary material for: Low-impedance tissue-device interface using homogeneously conductive hydrogels chemically bonded to stretchable bioelectronics
Source: Sci Adv. 2024 Mar 20;10(12):eadi7724. doi: 10.1126/sciadv.adi7724 (PMC10954228; doi:10.1126/sciadv.adi7724)
Supplement: Supplementary file 1 — Figs. S1 to S22 Legend for movie S1 [file sciadv.adi7724_sm.pdf]

Science Advances
AAAS

Supplementary Materials for

Low-impedance tissue-device interface using homogeneously conductive hydrogels chemically bonded to stretchable bioelectronics

Yoonsoo Shin *et al.*

Corresponding author: Sangkyu Lee, sangkyulee@snu.ac.kr; Dae-Hyeong Kim, dkim98@snu.ac.kr

Sci. Adv. **10**, eadi7724 (2024)
DOI: 10.1126/sciadv.adi7724

The PDF file includes:

Figs. S1 to S22
Legend for movie S1

Other Supplementary Material for this manuscript includes the following:

Movie S1

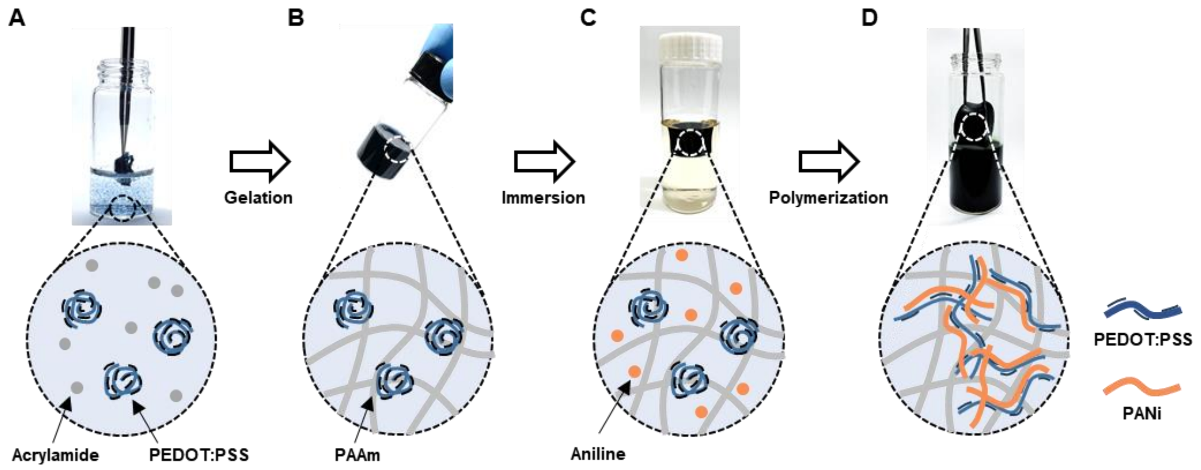

Fig. S1.

Steps for synthesizing a homogeneously conductive hydrogel. (**A**) Dispersion of freeze-dried PEDOT:PSS into the acrylamide solution. (**B**) Synthesis of the PEDOT:PSS-PAAm hydrogel. (**C**) Immersion of the PEDOT:PSS-PAAm hydrogel in the aniline solution. (**D**) Formation of the homogeneously conductive PEDOT:PSS-PANi-PAAm hydrogel.

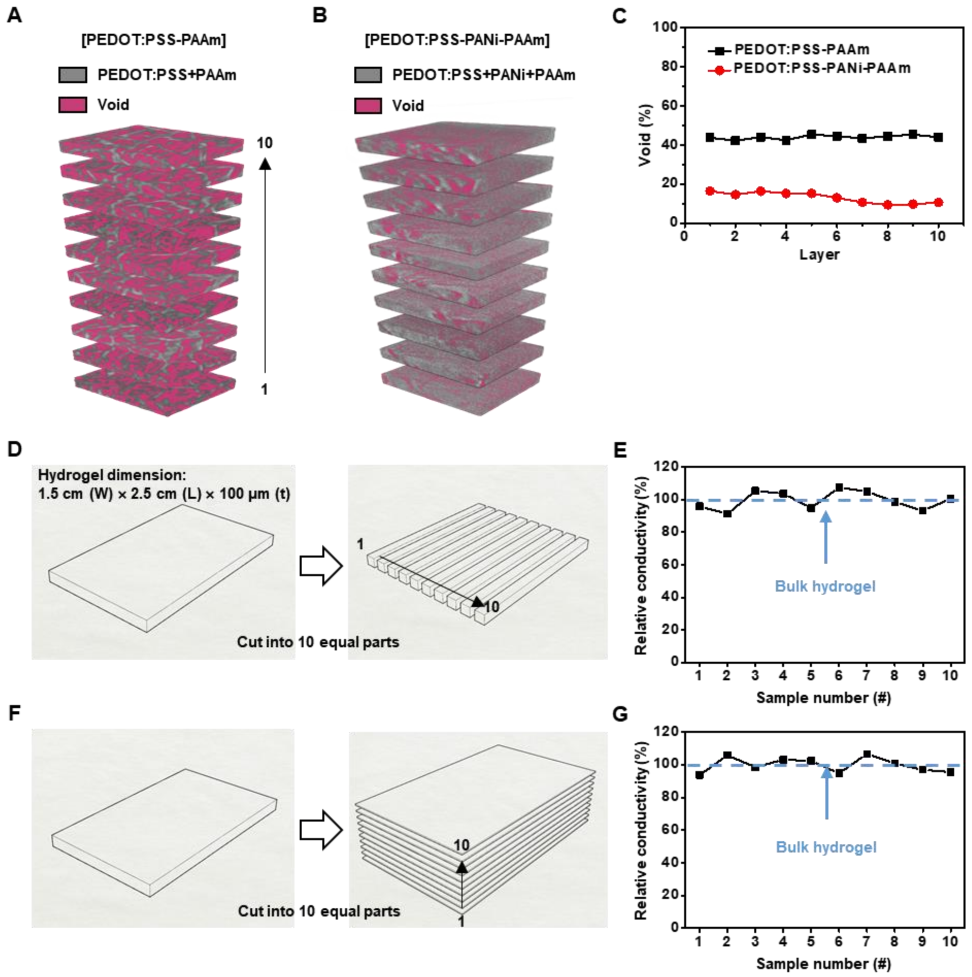

Fig. S2.

Hydrogel homogeneity analysis. (**A** and **B**) XRM images of the freeze dried PEDOT:PSS-PAAm (A) and PEDOT:PSS-PANi-PAAm (B). (**C**) Vertical quantity of voids within the PEDOT:PSS-PAAm and PEDOT:PSS-PANi-PAAm. (**D** and **E**) Longitudinally cutting the PEDOT:PSS-PANi-PAAm hydrogel (D) and the conductivity variation of each sample resulting from this vertical cutting (E). (**F** and **G**) Cutting the PEDOT:PSS-PANi-PAAm hydrogel in the thickness direction (F) and the conductivity variation of each layer resulting from this vertical cutting (G).

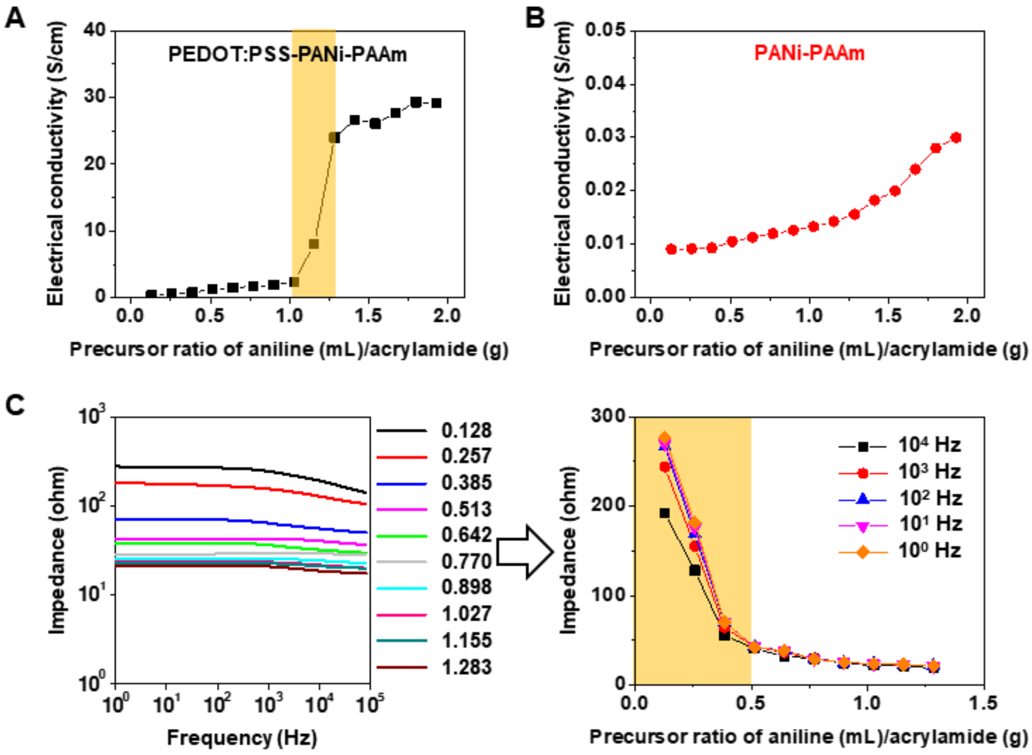

Fig. S3.

Electrical conductivity and impedance of hydrogels functionalized with different aniline concentration. (**A** and **B**) Electrical conductivity of PEDOT:PSS-PANi-PAAm (A) and PANi-PAAm hydrogels (B) prepared by varying precursor ratio of aniline (mL) to acrylamide (g). (**C**) Impedance of PEDOT:PSS-PANi-PAAm hydrogel, prepared by varying aniline concentrations, as a function of frequency. The numbers indicate the precursor ratio of aniline (mL) to acrylamide (g).

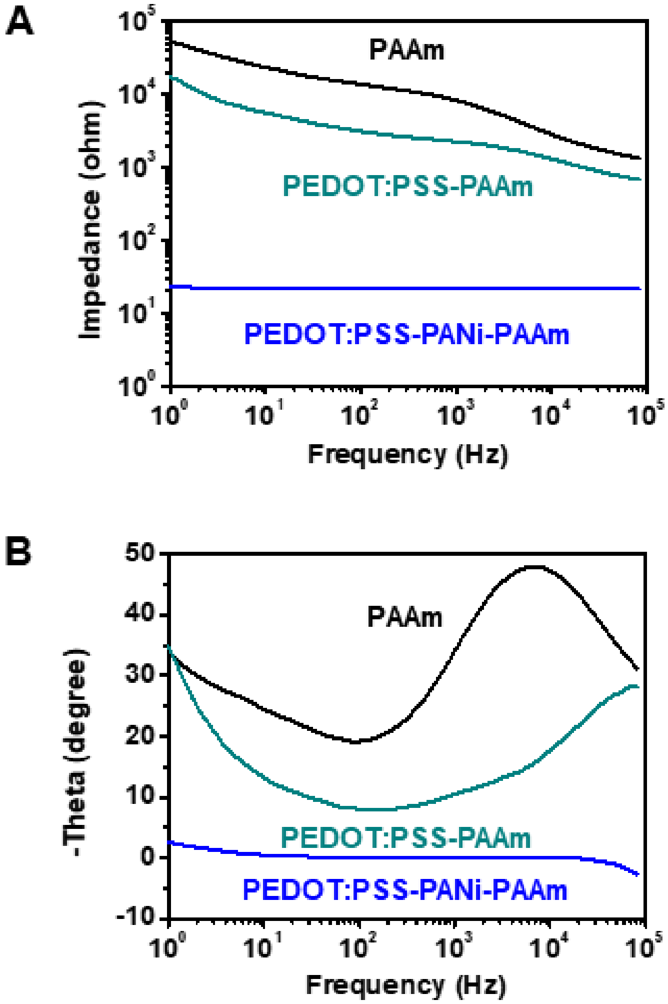

Fig. S4.

Electrochemical impedance spectroscopy results for PAAm, PEDOT:PSS-PAAm, and PEDOT:PSS-PANi-PAAm hydrogels. (**A** and **B**) Impedance (A) and phase angle (B) variations with respect to frequency.

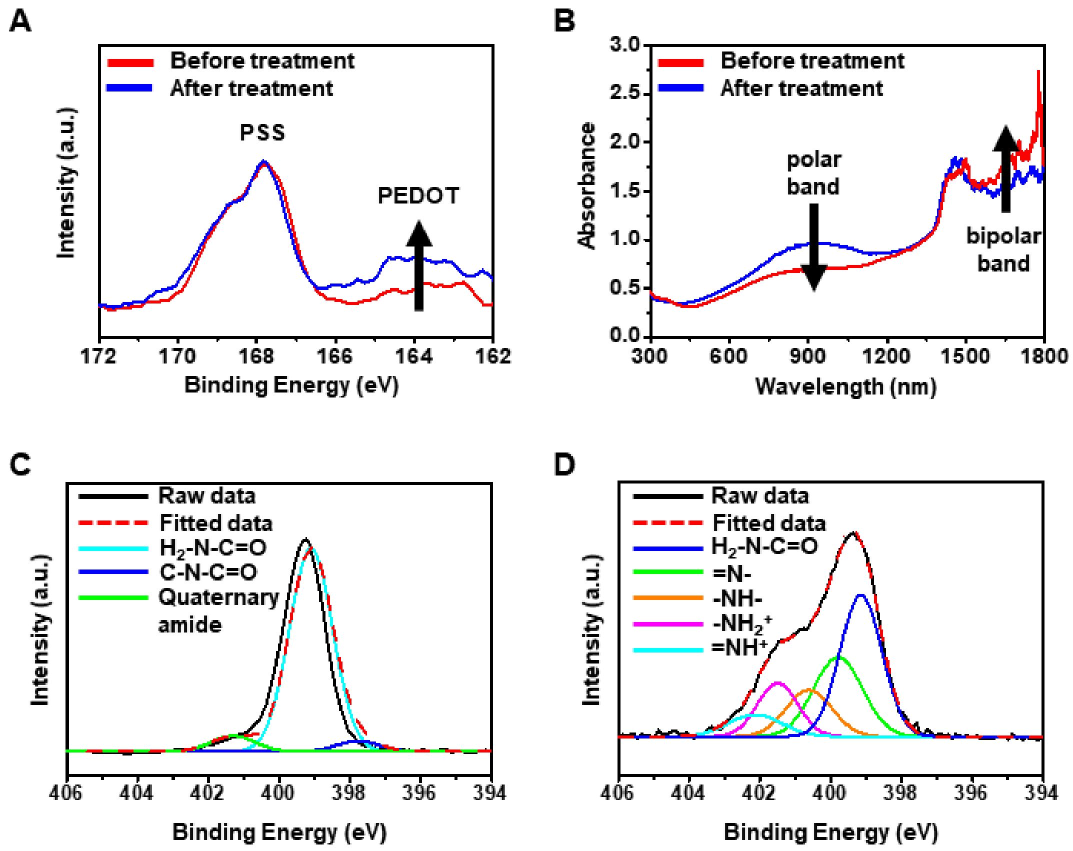

Fig. S5.

XPS and UV-vis-NIR spectroscopy results for PEDOT:PSS-PAAm and PEDOT:PSS-PANi-PAAm hydrogels. (**A** and **B**) S2p XPS spectra (A) and UV-vis-NIR absorbance spectra (B) of PEDOT:PSS-PAAm hydrogel, before and after treatment with aniline solution. (**C** and **D**) N1s XPS spectra of PEDOT:PSS-PAAm hydrogel (C) and PEDOT:PSS-PANi-PAAm hydrogel.

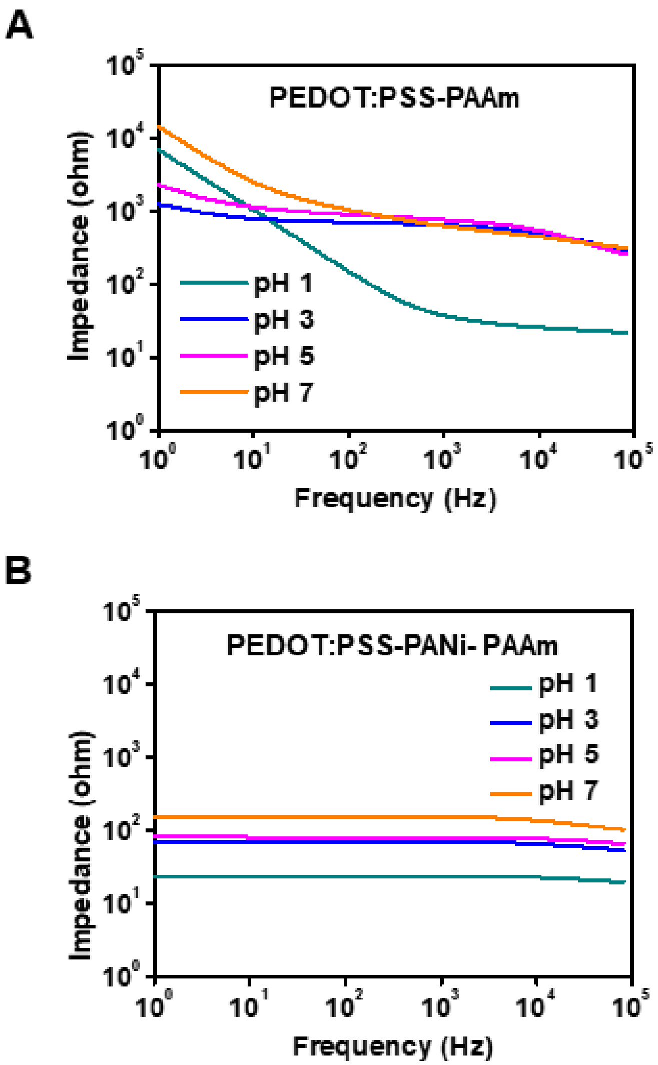

Fig. S6.

Impedance of PEDOT:PSS-PAAm and PEDOT:PSS-PANi-PAAm hydrogels. (**A** and **B**) Impedance of PEDOT:PSS-PAAm (A) and PEDOT:PSS-PANi-PAAm hydrogels at various pH values (B).

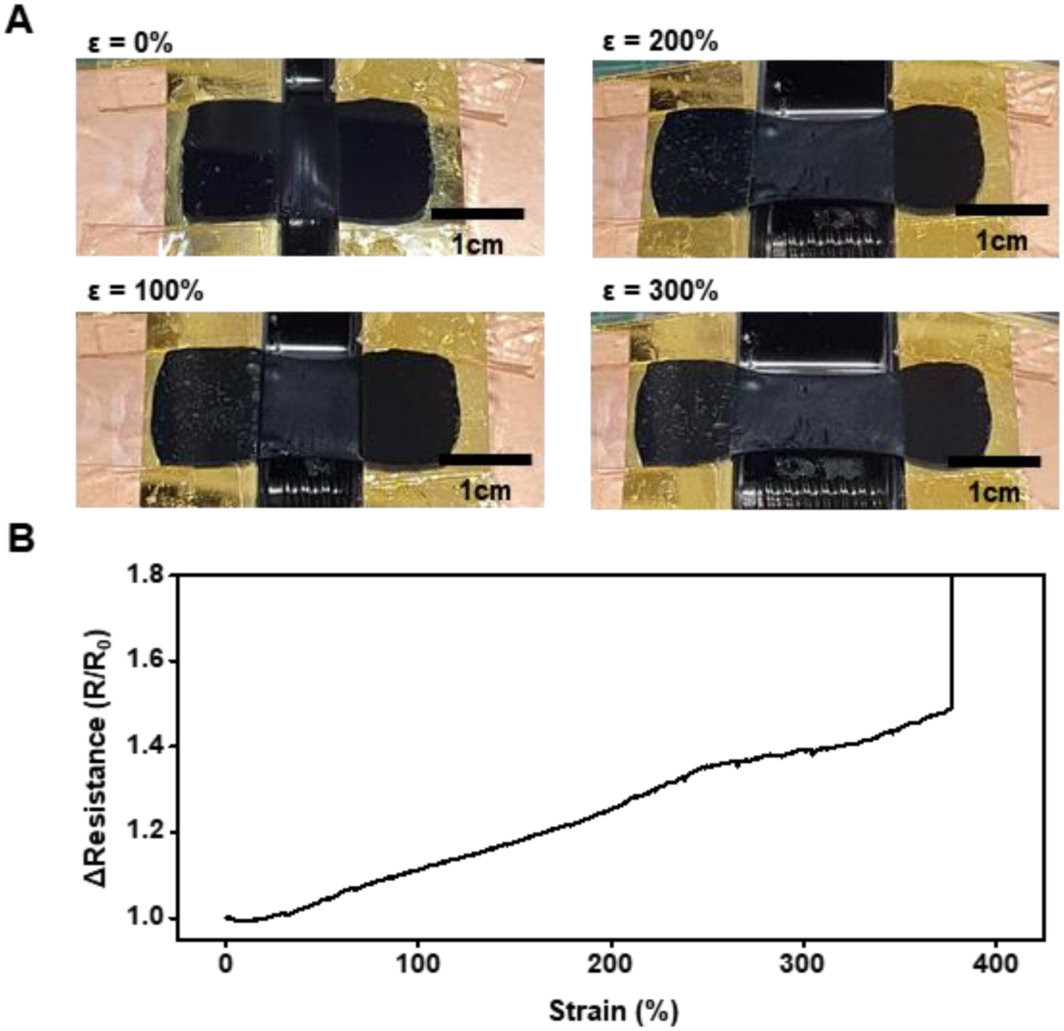

Fig. S7.

Stretching test of PEDOT:PSS-PANi-PAAm hydrogel. (**A**) Images of the PEDOT:PSS-PANi-PAAm hydrogel at different stains of 0%, 100%, 200%, and 300%. (**B**) Resistance variation during the stretching of the PEDOT:PSS-PANi-PAAm hydrogel.

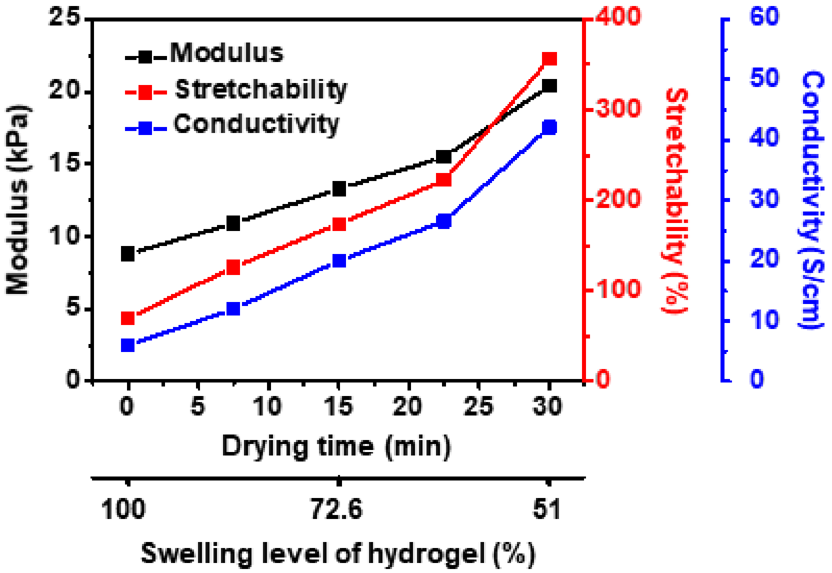

Fig. S8.

Electrical and mechanical properties of PEDOT:PSS-PANi-PAAm hydrogel in response to changes in its swelling level.

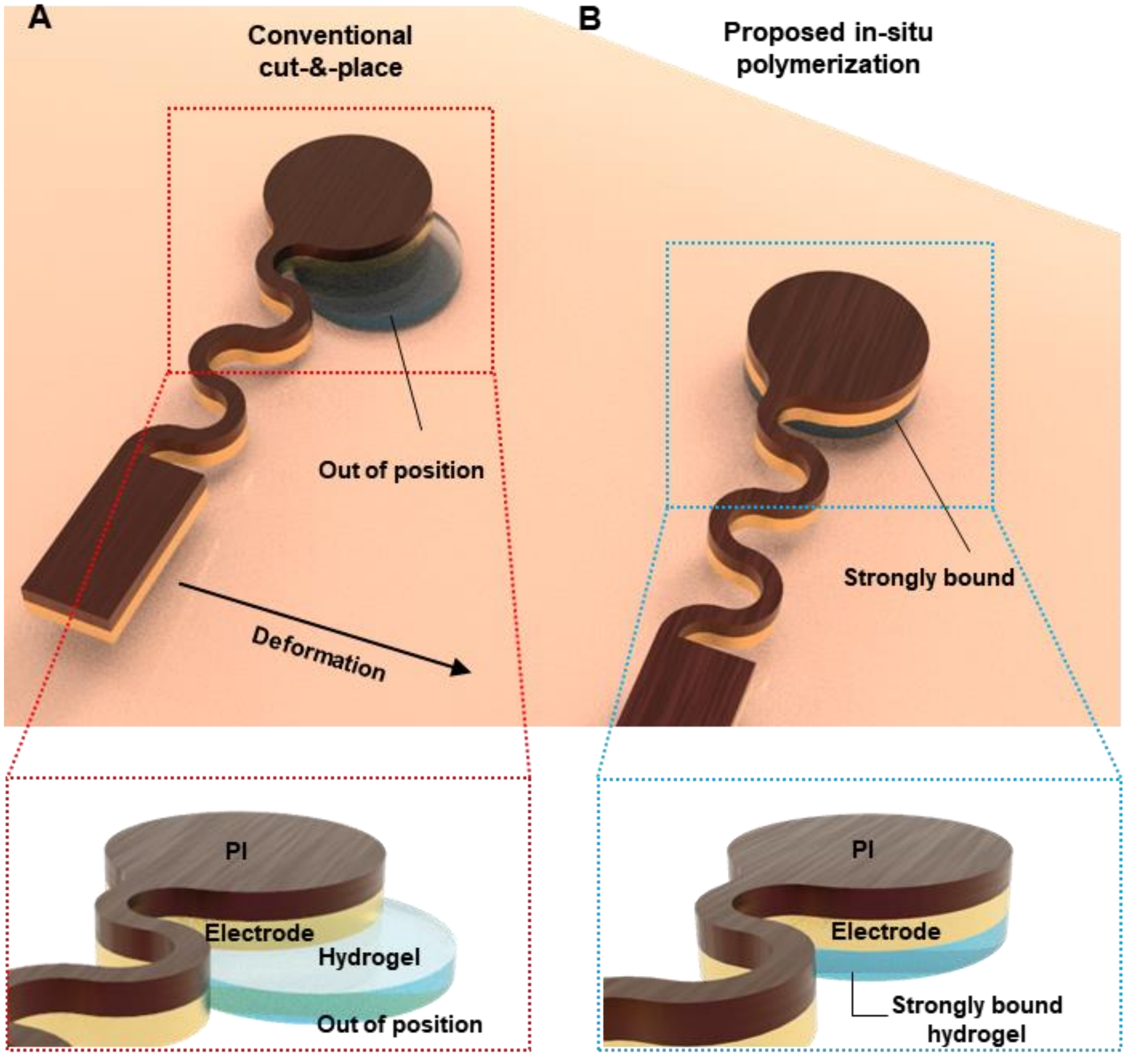

Fig. S9.

Schematic illustration of hydrogel integrated on an electrode. (**A**) Hydrogel film located on an electrode through conventional "cut-&-place" method. (**B**) *In situ* polymerized hydrogel on an electrode.

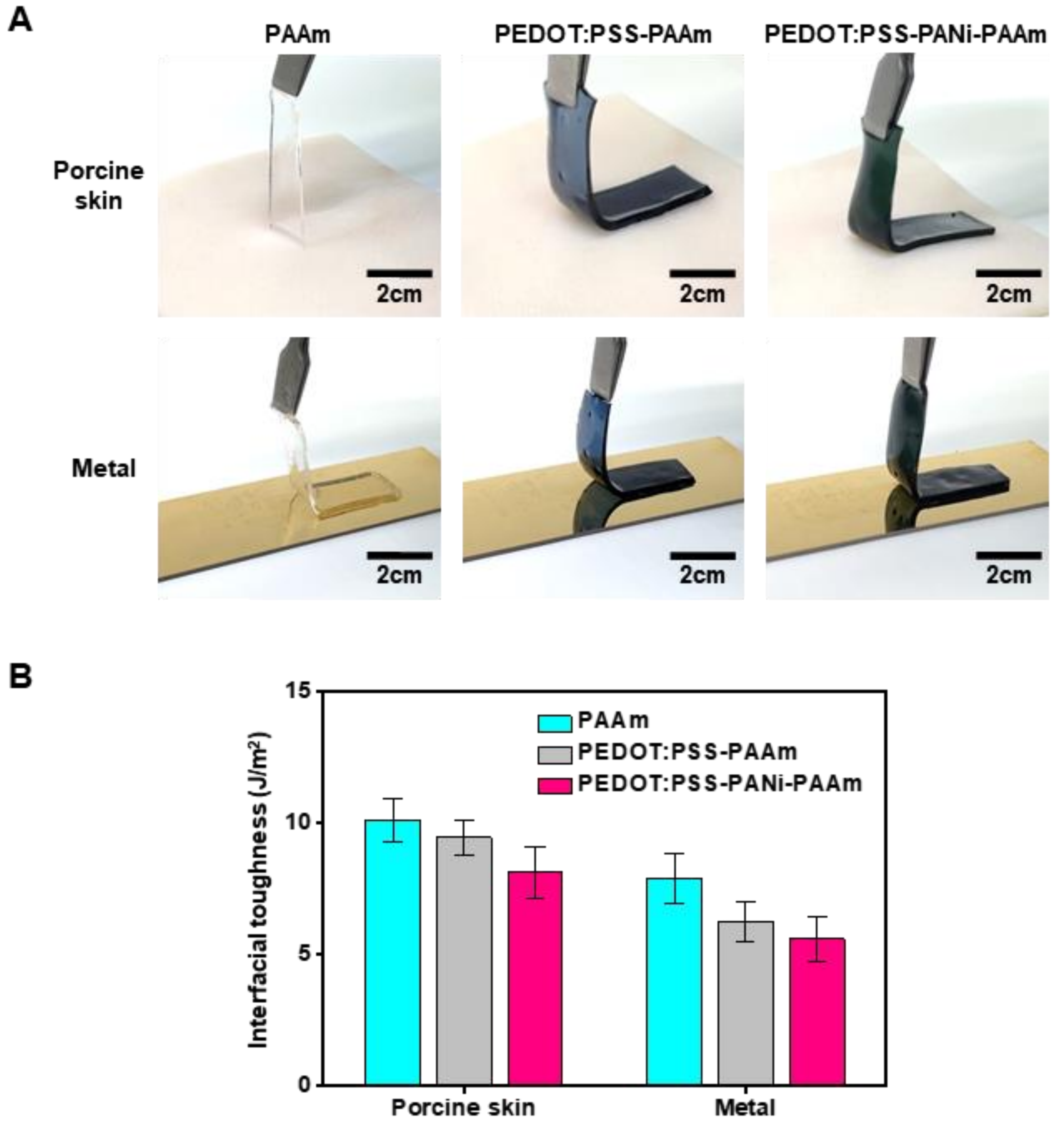

Fig. S10.

Adhesion of hydrogels applied to porcine skin and metal ('cut-and-place' method). (**A**) Images of peeling test conducted on PAAm, PEDOT:PSS-PAAm, and PEDOT:PSS-PANi-PAAm hydrogels from both porcine skin and metal substrate. (**B**) Interfacial toughness of the hydrogels on porcine skin and metal substrate.

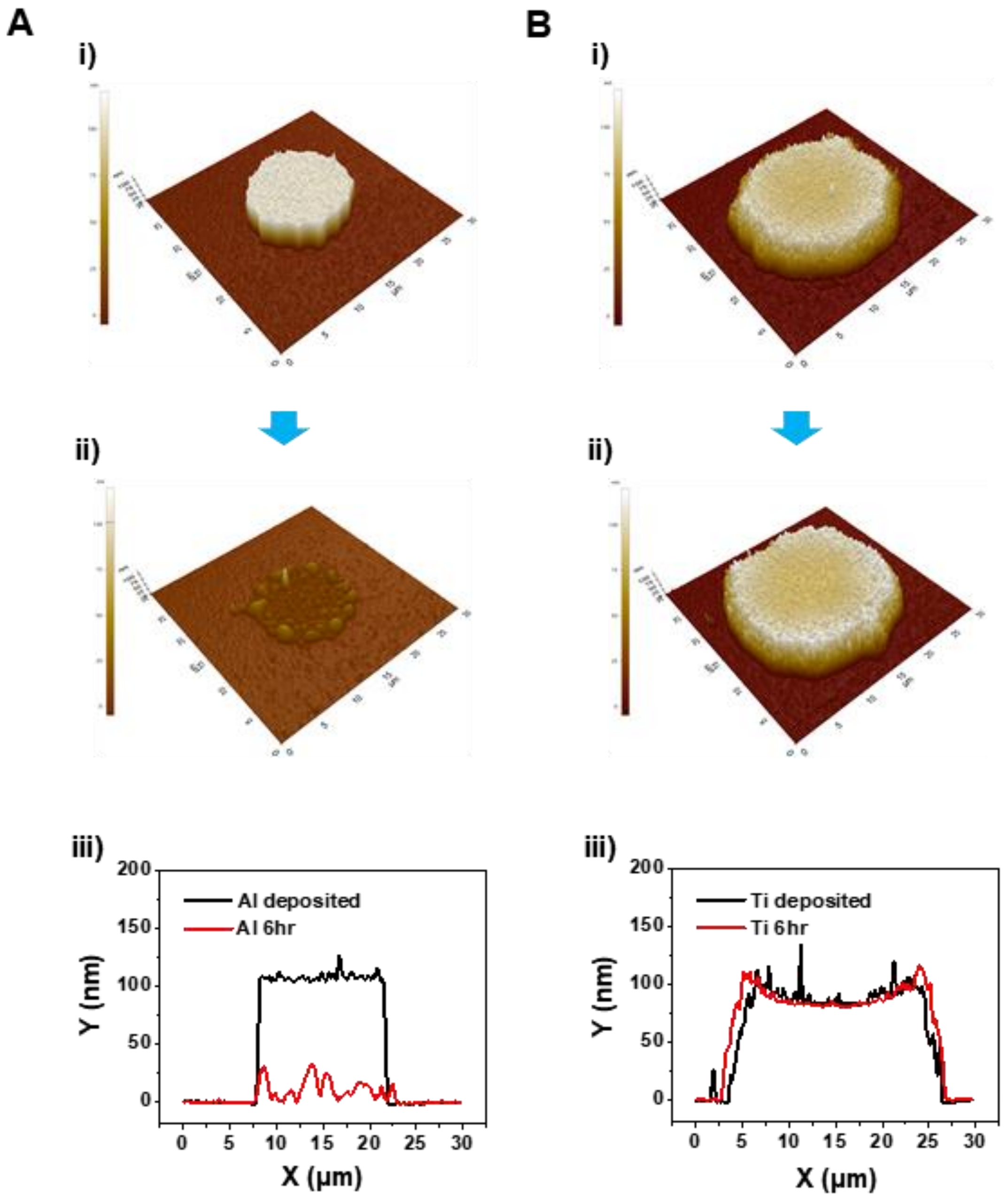

Fig. S11.

Reactivity of metals in acidic condition. (**A** and **B**) 3D atomic force microscopy images (i, ii) and surface roughness profiles (iii) of Al (A) and Ti (B). The upper images (i) correspond to the as-deposited metals, while the lower images (ii) correspond to the metal thin-films immersed in a 1N HCl bath for 6 h.

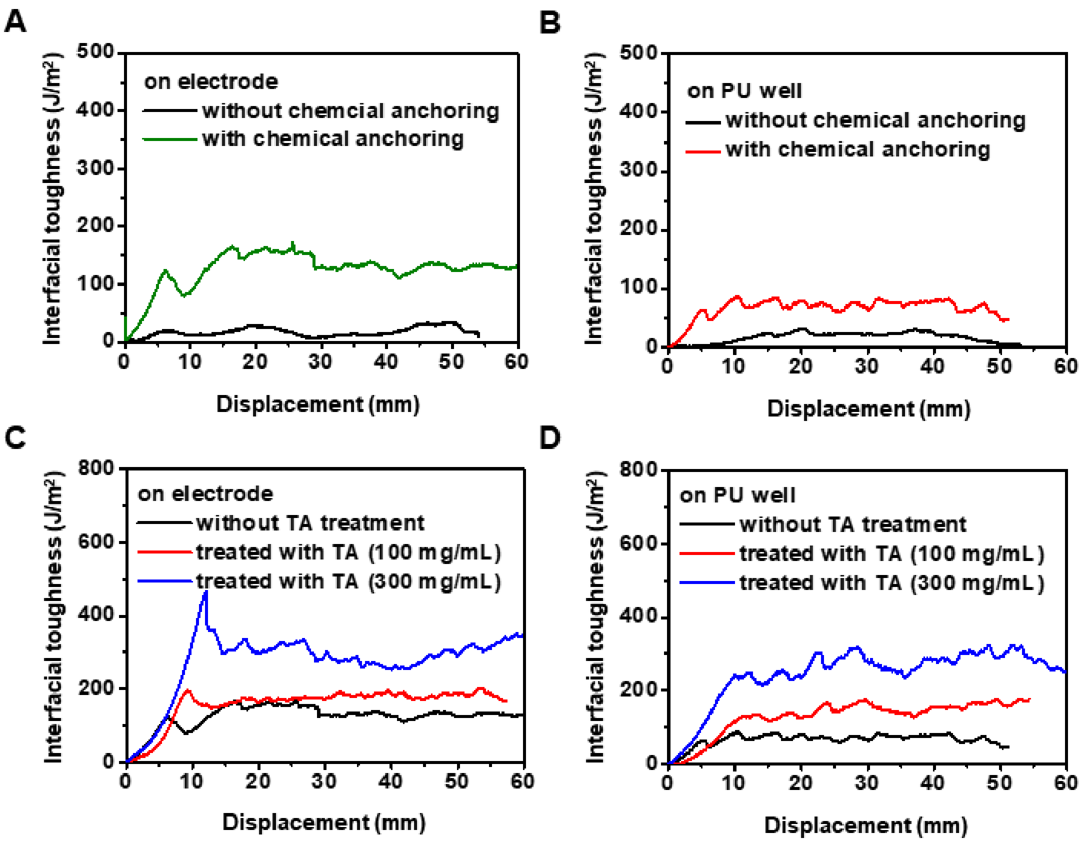

Fig. S12.

Adhesion test on PEDOT:PSS-PANi-PAAm hydrogel. (**A** and **B**) 90° peeling of PEDOT:PSS-PANi-PAAm hydrogel from the electrode (A) and PU well (B), with and without chemical anchoring. (**C** and **D**) 90° peeling of PEDOT:PSS-PANi-PAAm hydrogel from the chemically integrated electrode (C) and PU well (D), with and without TA treatment.

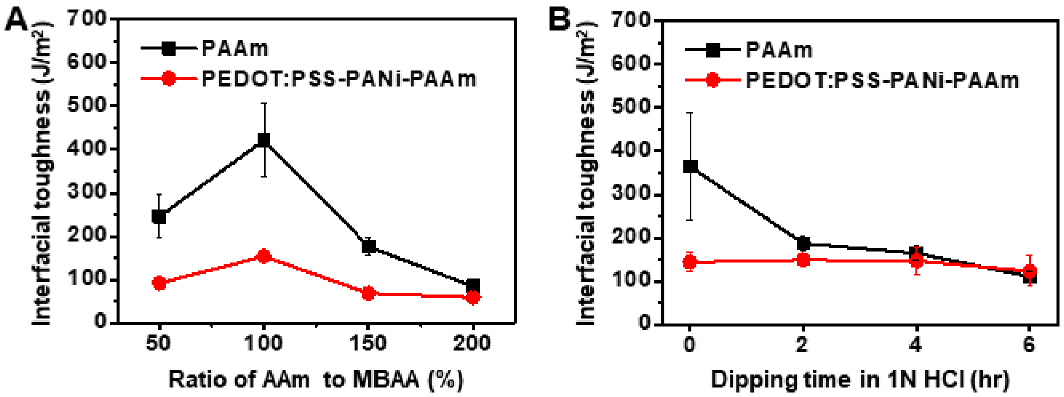

Fig. S13.

Optimization of hydrogel precursor ratios for strong bonding and stability of hydrogels in acidic environment. (**A**) Optimization of PAAm and PEDOT:PSS-PANi-PAAm hydrogels via adjustments in the acrylamide to MBAA precursor ratio. (**B**) Evaluation of hydrogel stability under acidic conditions (1N HCl) relative to immersion duration.

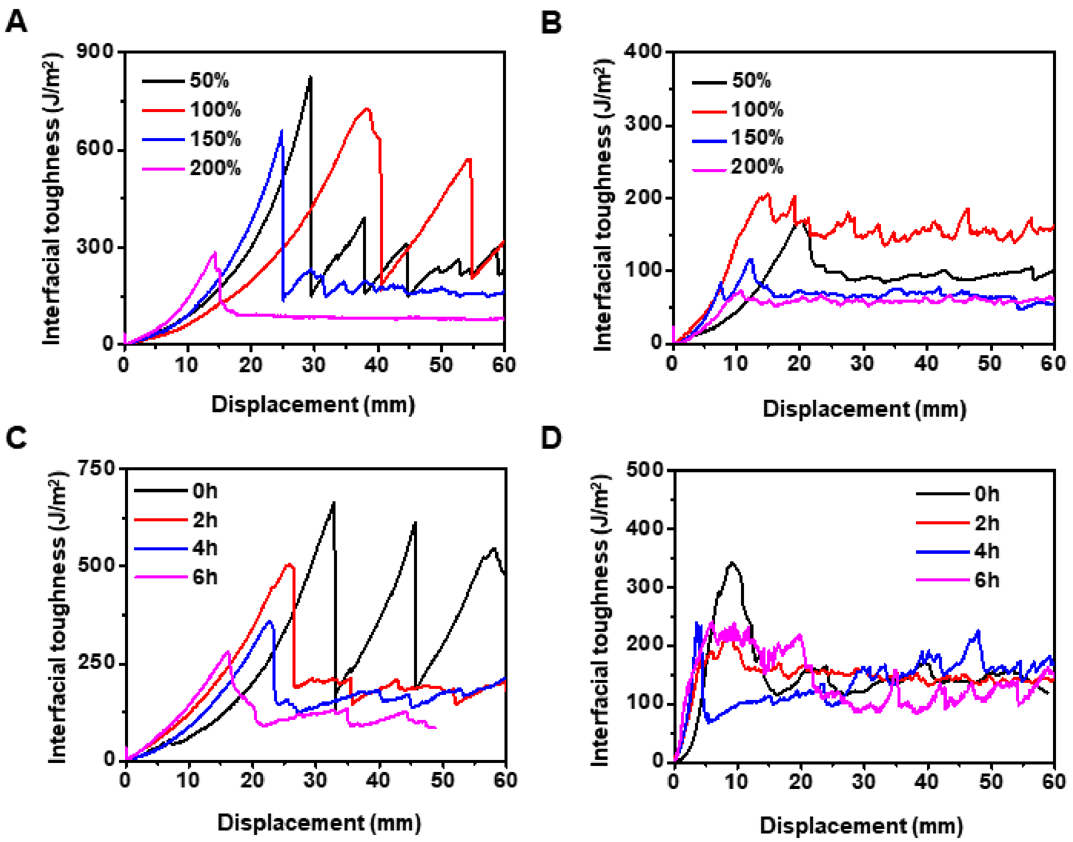

Fig. S14.

Adhesion test on PAAm and PEDOT:PSS-PANi-PAAm hydrogels. (**A** and **B**) Interfacial toughness of PAAm (A) and PEDOT:PSS-PANi-PAAm hydrogels (B) prepared by varying the ratio of acrylamide to MBAA at 50%, 100%, 150%, and 200%. (**C** and **D**) Changes in interfacial toughness of the PAAm (C) and PEDOT:PSS-PANi-PAAm hydrogels (D) formed on a chemically-treated substrate upon immersion in 1N HCl solution with increasing immersion time up to 6h.

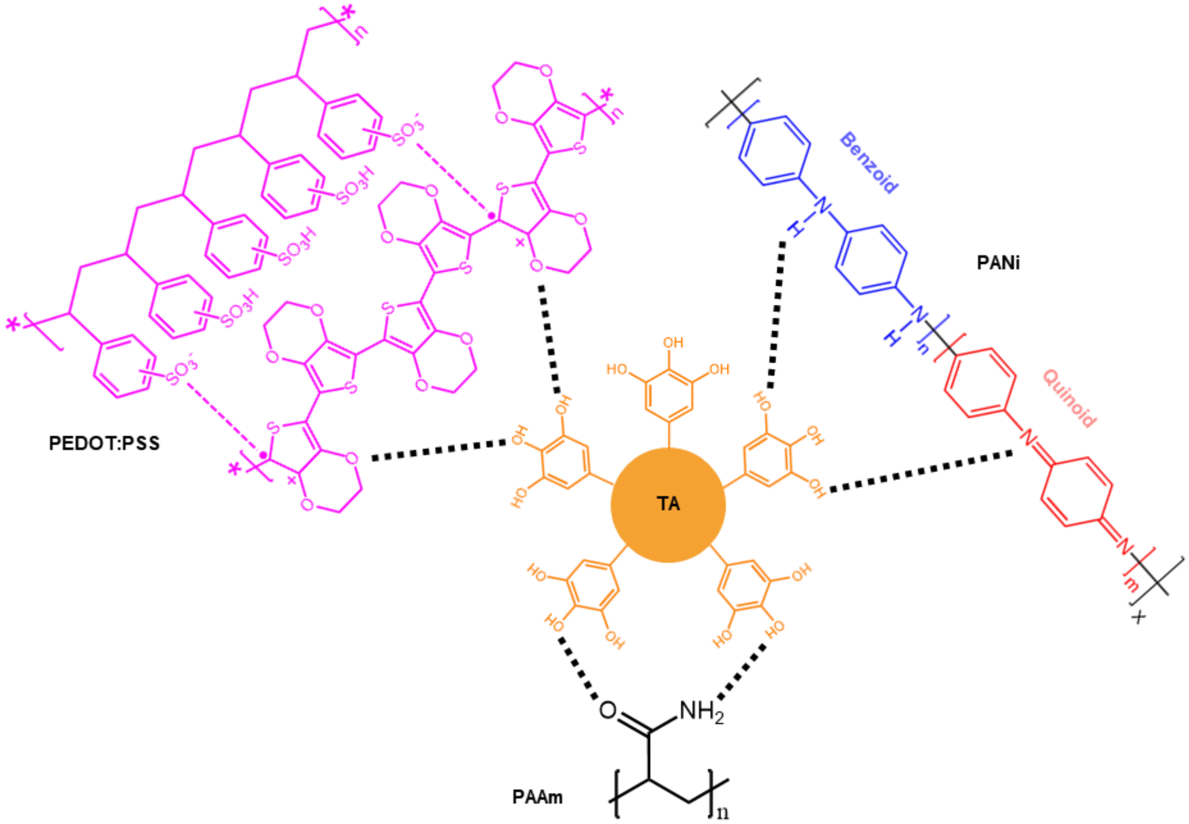

Fig. S15.

Hydrogen bonding between a TA molecule and others including PEDOT:PSS, PANi, and PAAm within the conductive hydrogel.

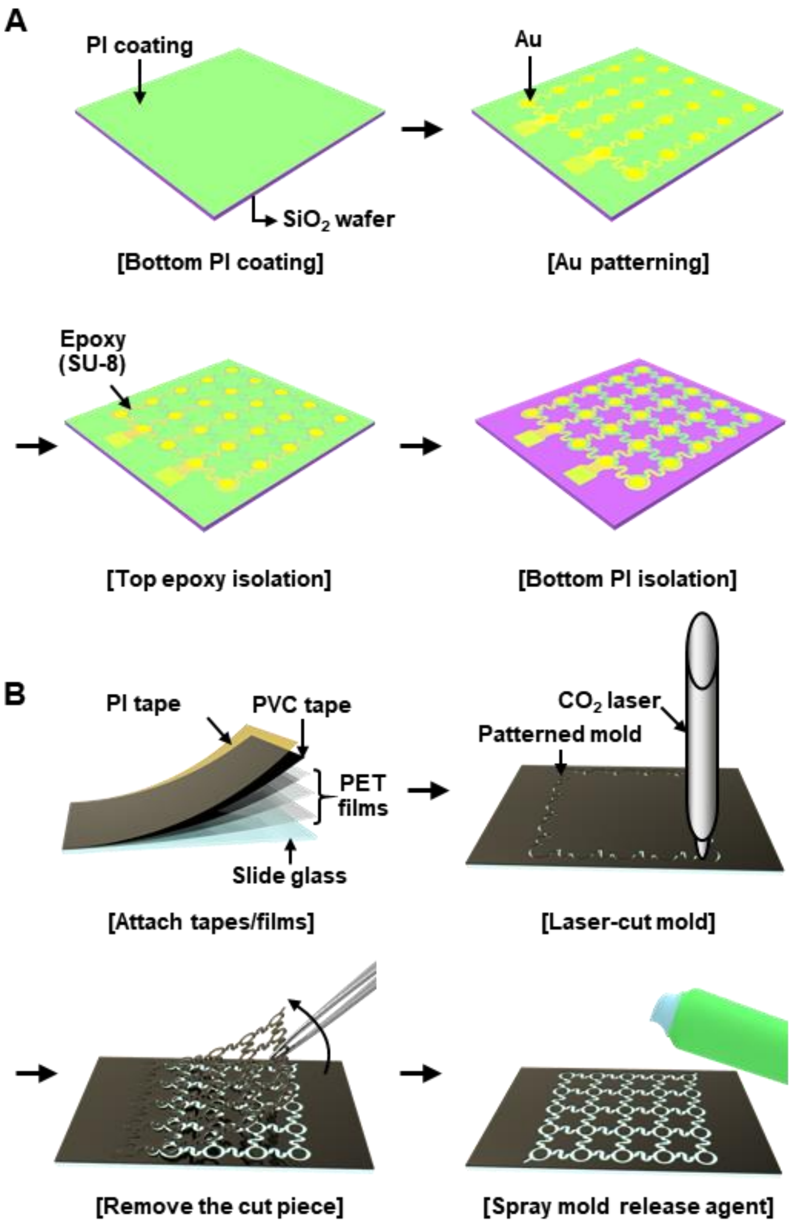

Fig. S16.

Illustration depicting detailed fabrication steps for stretchable bioelectronics and elastic PU wells. (**A**) Stretchable bioelectronics. (**B**) Elastic PU wells.

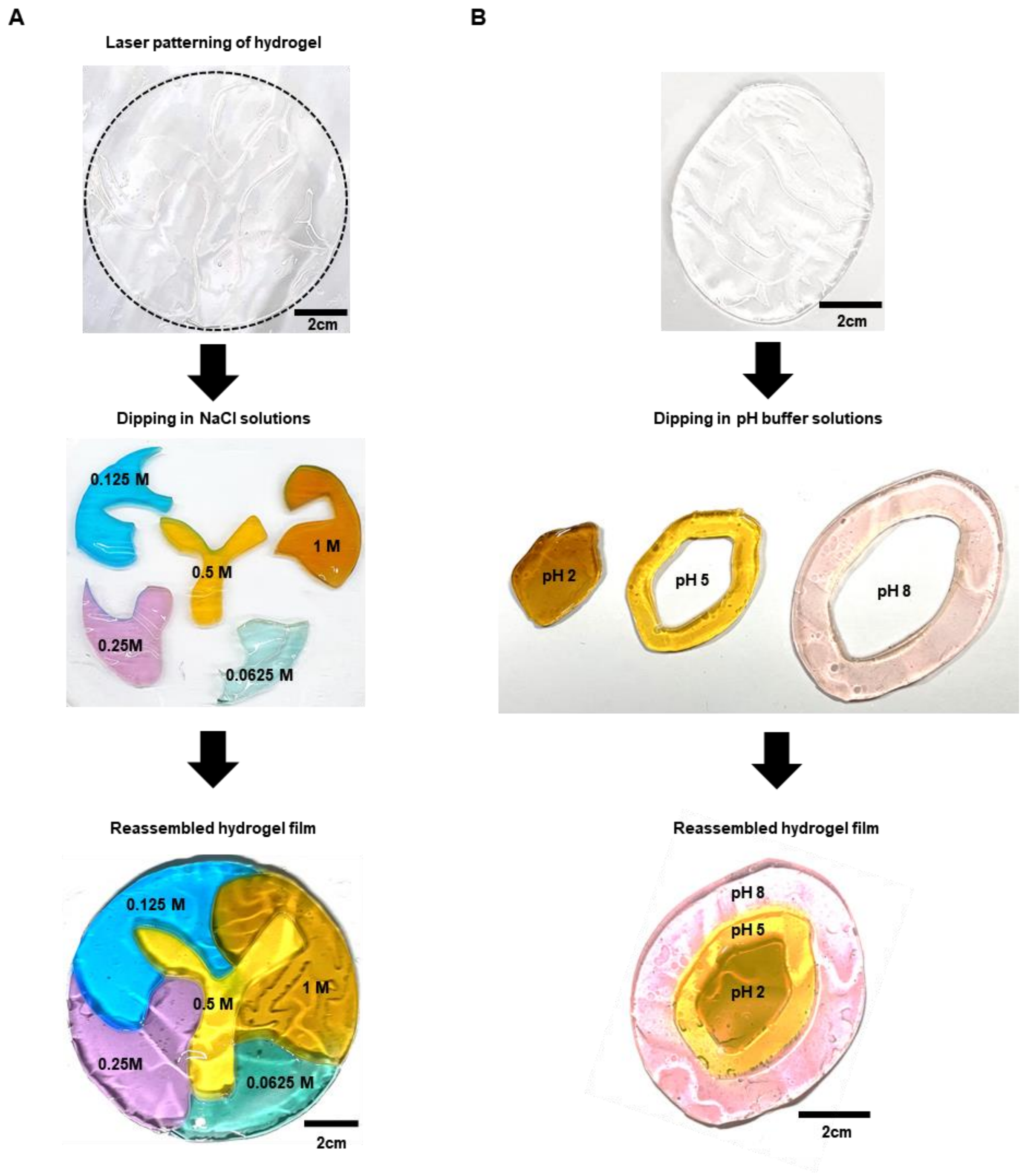

Fig. S17.

Preparation steps for artificial skin. (**A** and **B**) Artificial skin made of hydrogel with different ionic concentrations (A) and pH levels (B).

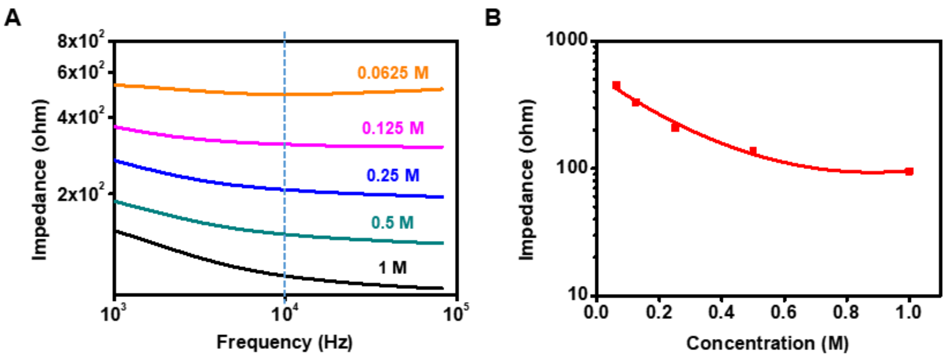

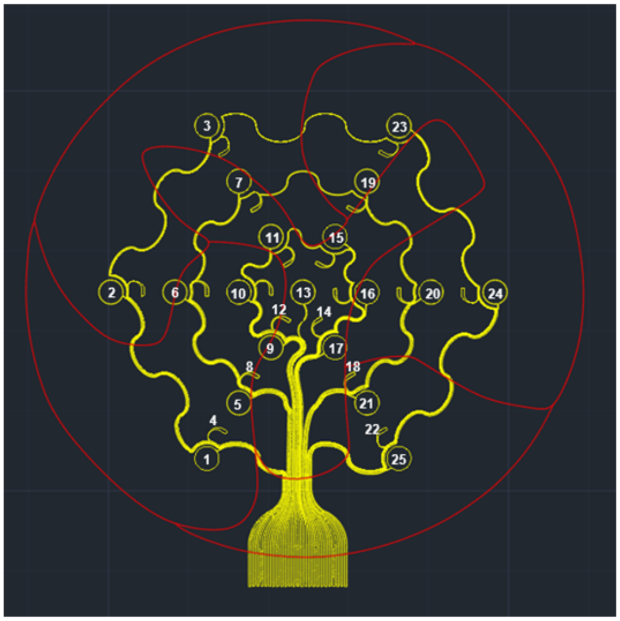

D

Position	Impedance @ 10 KHz
1,5	205.6
5,9	197.7
9,13	157
13,15	168.3
15,19	112
19,23	105.7
2,6	252
6,10	221.5
10,13	159.4
13,16	112.4
16,20	92.9
20,24	90.95
3,7	179.5
7,11	147.5
11,13	145.7
13,17	147.1
17,21	221.9
21,25	443.3

Fig. S18.

Measurement of impedance using multichannel patch. (**A**) Impedance results. (**B**) Calibration curves. (**C**) Measurement points of impedance. (**D**) Impedance values at the positions.

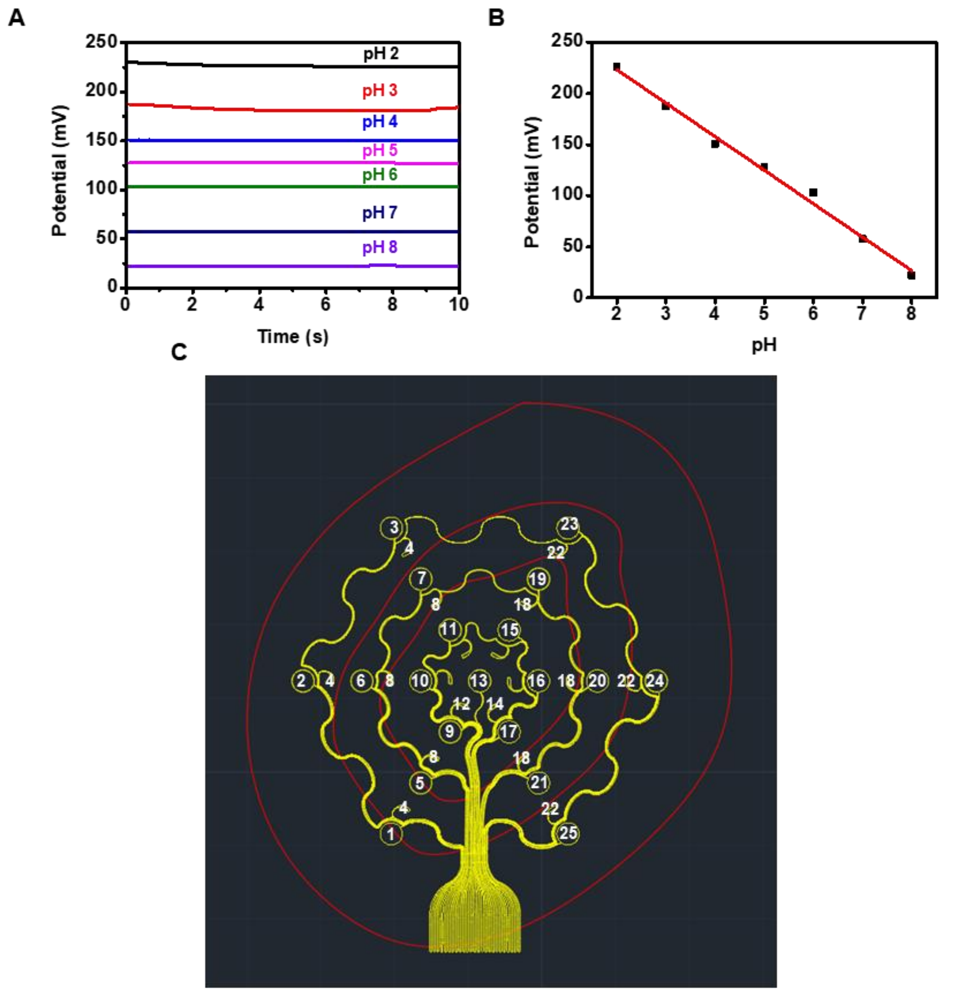

D

Position	Potential (mV)	Position	Potential (mV)	Position	Potential (mV)
1,4	70.9	2,4	22.5	3,4	27.8
4,5	152.9	4,6	51.4	4,7	50.3
5,8	199	6,8	204	7,8	155.5
8,9	228.8	8,10	212.3	8,11	218.8
9,12	213.5	10,12	218.6	11,12	222.5
12,13	219.4	12,13	222.6	12,13	215.1
13,14	233	13,14	262.9	13,14	230.8
14,15	229.2	14,16	229.3	14,17	223.7
15,18	222.1	16,18	217.8	17,18	187.4
18,19	213.7	18,20	180	18,21	121.1
19,22	113.7	20,22	129.1	21,22	108.6
22,23	100.7	22,24	93	22,25	33.1

Fig. S19.

Measurement of pH using multichannel patch. (**A**) Measured potential between PANi-PAAm hydrogel and Ag/AgCl electrode. (**B**) Calibration curves. (**C**) Measurement points of pH. (**D**) pH levels at the positions.

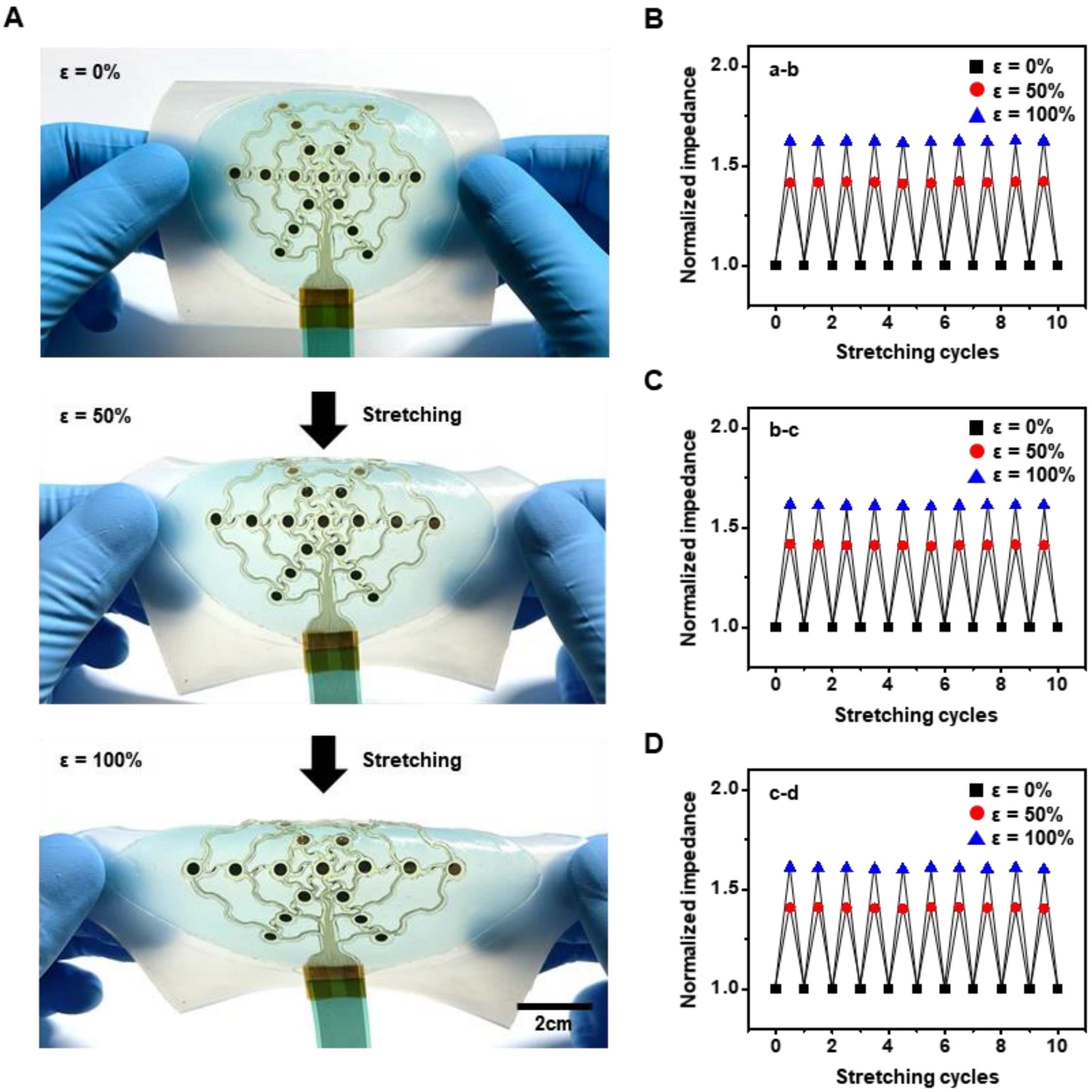

Fig. S20.

Impedance mapping during stretching deformation. (**A**) Images of the stretchable multichannel sensor array on the artificial tissue at various stretching levels. (**B** to **D**) Impedance mapping at different points during stretching cycles: position a-b (B), position b-c (D), and position c-d (D).

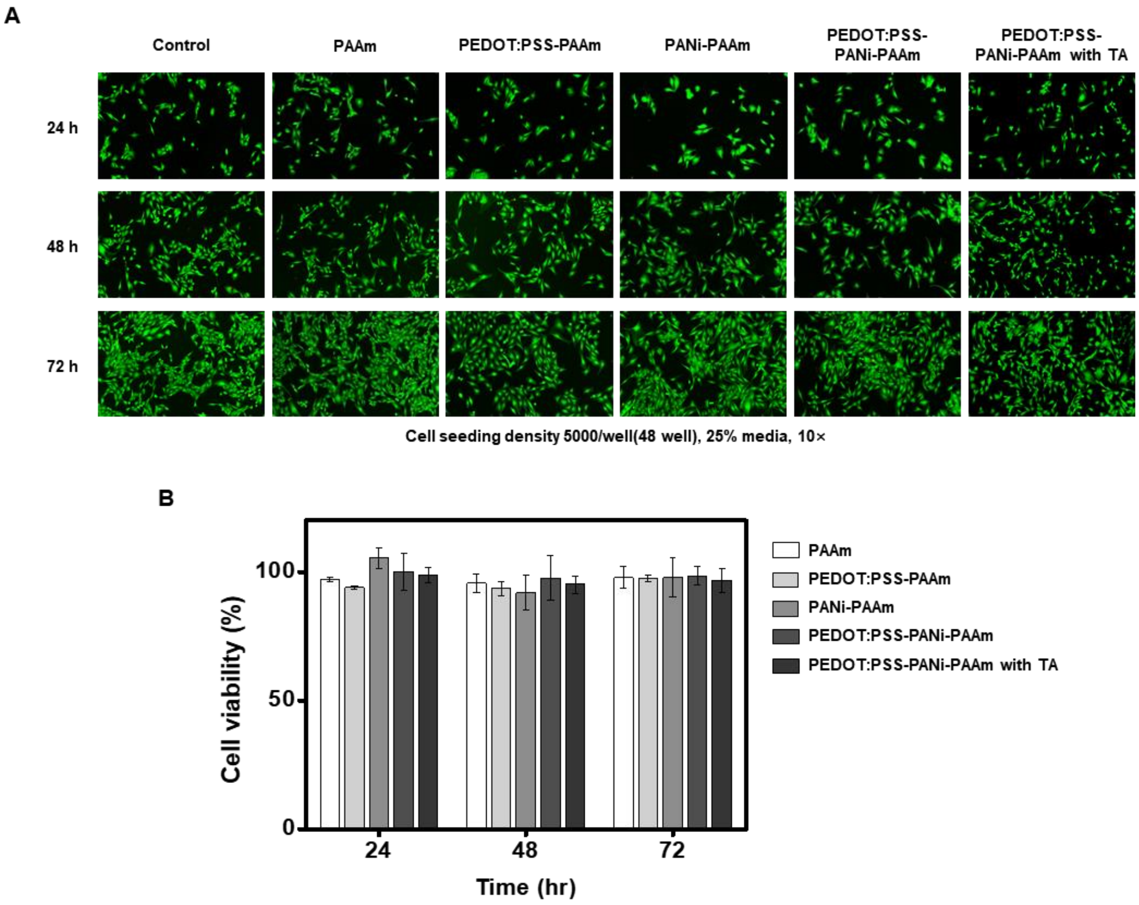

Fig. S21.

Biocompatibility of the conductive hydrogel. (**A**) Cell proliferation results up to 72 h. (**B**) Cell viability results up to 72h.

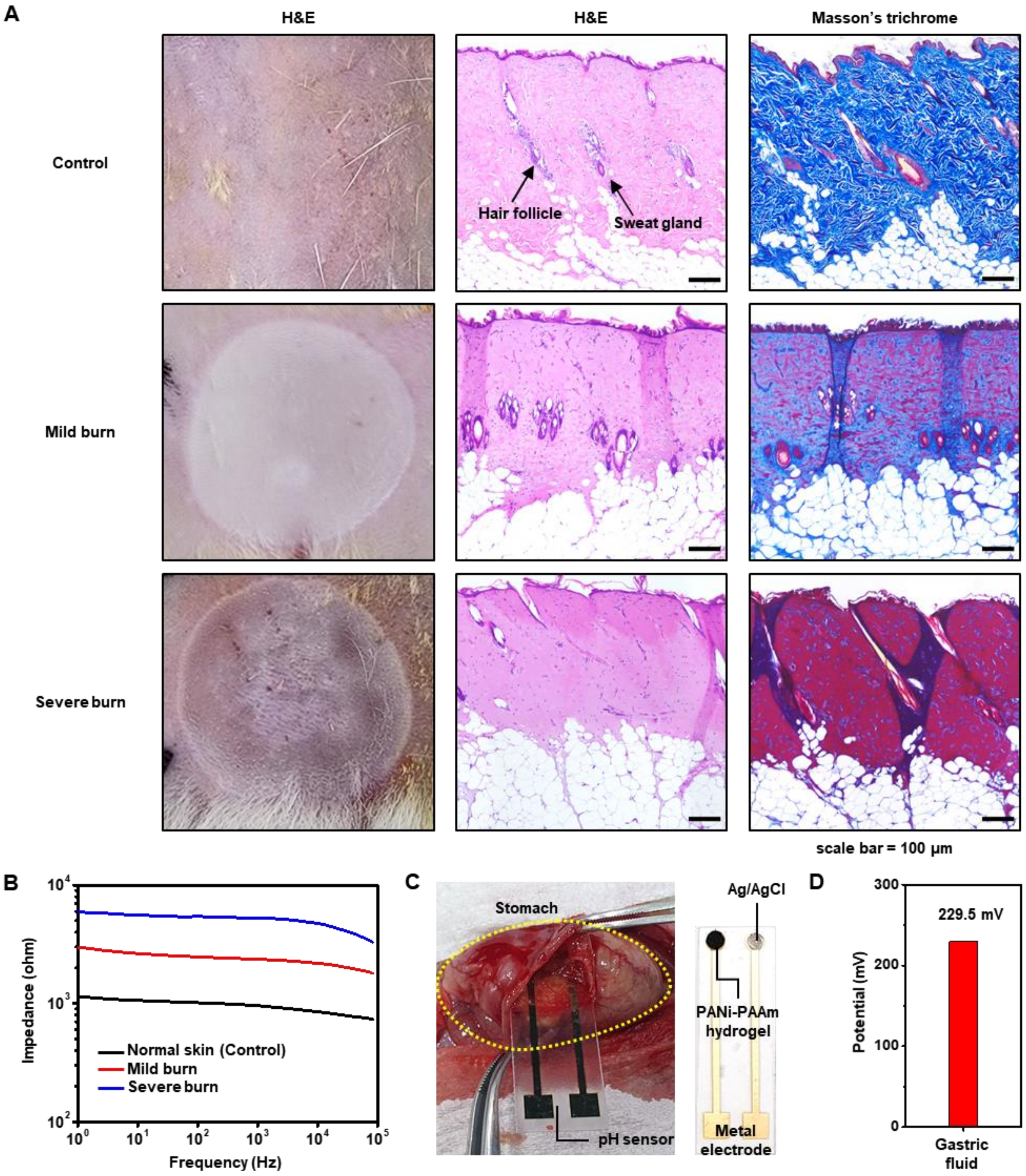

Fig. S22.

***In vivo* impedance and pH measurement demonstration.** (**A**) Skin burn wound models of control, mild burn, and severe burn. The images show skin surface (left), and hematoxylin and eosin (H&E)-stained (middle) and Masson's trichrome-stained cross-sections of rat skin samples (right). (**B**) *In vivo* measurement of skin impedance in rat after skin burn injury. (**C**) Images of the stomach and the inserted pH sensor (left), as well as the pH sensor with a PANi-PAAm hydrogel electrode and an Ag/AgCl electrode (right). (**D**) *In vivo* measurement of gastric fluid pH in a living rat.

Movie S1.

Stretching of the stretchable multichannel sensor array placed on the artificial tissue model.
